# Synthesis and Antifungal Activity of Novel Myrtenal-Based 4-Methyl-1,2,4-triazole-thioethers

**DOI:** 10.3390/molecules22020193

**Published:** 2017-01-24

**Authors:** Gui-Shan Lin, Wen-Gui Duan, Lin-Xiao Yang, Min Huang, Fu-Hou Lei

**Affiliations:** 1 School of Chemistry and Chemical Engineering, Guangxi University, Nanning 530004, Guangxi, China; gxlinguas@sina.com (G.-S.L.); gslin@gxu.edu.cn (L.-X.Y.); gaominhuang@mail.gxu.cn (M.H.); 2 Guangxi Key Laboratory of Chemistry and Engineering of Forest Products, Nanning 530008, Guangxi, China; dwg105@gxu.edu.cn

**Keywords:** α-pinene, myrtenal, 1,2,4-triazole-thioether, antifungal activity

## Abstract

A series of novel myrtenal derivatives bearing 1,2,4-triazole moiety were designed and synthesized by multi-step reactions in an attempt to develop potent antifungal agents. Their structures were confirmed by using UV-vis, FTIR, NMR, and ESI-MS analysis. Antifungal activity of the target compounds was preliminarily evaluated by the in vitro method against *Fusarium oxysporum* f. sp. *cucumerinum*, *Physalospora piricola*, *Alternaria solani*, *Cercospora arachidicola*, and *Gibberella zeae* at 50 µg/mL. Compounds **6c** (R = *i*-Pr), **6l** (R = *o*-NO_2_ Bn), and **6a** (R = Et) exhibited excellent antifungal activity against *P. piricola* with inhibition rates of 98.2%, 96.4%, and 90.7%, respectively, showing better or comparable antifungal activity than that of the commercial fungicide azoxystrobin with a 96.0% inhibition rate, which served as a positive control.

## 1. Introduction

Myrtenal—a natural bicyclic monoterpene containing an aldehyde group, a carbon-carbon double bond, and a four-membered ring—was found in essential oils obtained from medicinal plants such as *Artemisia douglasiana* [1], *Ferula hermonis* [2], *Lepidium meyenii* [3], *Otacanthus* [4], and *Mentha haplocalyx* [5]. It can also be conveniently prepared by the regioselective oxidation reaction of α-pinene, the main component of turpentine oil, which is an abundant natural product. As a secondary metabolite associated with medicinal essential oils, myrtenal has received growing interest in the investigation for its bioactive properties and was found to exhibit a broad spectrum of activities, such as anticancer [5,6,7], antimicrobial [1], acetylcholinesterase inhibitory [8], antimalarial [9], black bean aphid repellent [10], and bark beetle semiochemical [11,12] activities. Also, some myrtenal-based amide derivatives were synthesized by Nomura et al., and they were found to display insecticidal, mosquito repellent, and herbicidal activities [13,14,15]. Based on its bioactive property and chemical reactivity, myrtenal deserves further study for pharmaceutical or agrochemical use. On the other hand, 1,2,4-triazole derivatives were reported to possess diverse biological activities, such as antimicrobial [16,17], antifungal [18], antitumor [19], anti-inflammatory [17,20], antituberculosis [21], and herbicidal [22] properties. In continuation of our interest in the bioactive properties of natural product-based compounds [23,24,25,26,27,28], a series of novel myrtenal-based 4-methyl-1,2,4-triazole-thioethers were designed and synthesized by integrating bioactive 1,2,4-triazole and thioether moieties into the skeleton of myrtenal converted from α-pinene. Structural characterization and antifungal evaluation of all the title compounds were carried out as well.

## 2. Results and Discussion

### 2.1. Synthesis and Characterization

**Scheme 1:**
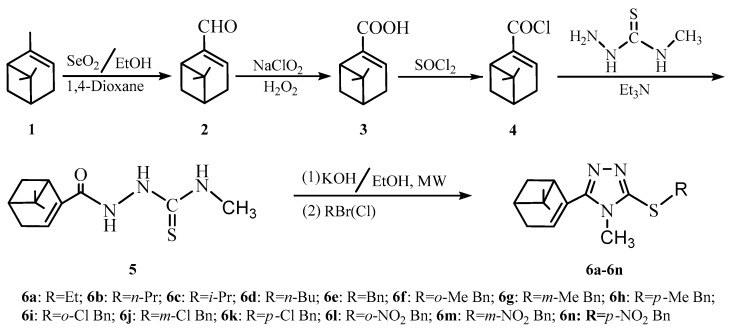
Synthesis of myrtenal-based 4-methyl-1,2,4-triazole-thioethers **6a**–**6n**.

As illustrated in Scheme 1, the key intermediate myrtenal was prepared by regioselective oxidation of α-pinene using the product from the reaction of selenium dioxide with anhydrous ethanol as oxidant and 1,4-dioxane as solvent in a 70% yield. This reaction could be driven by removing low boiling products of ethanol and water from the reacting system, providing a good yield in a short reaction time even in the absence of the pro-oxidant oxygen employed by the reported methods [11,12]. Myrtenic acid was prepared by further oxidation of myrtenal using a combination of NaClO_2_-H_2_O_2_ as oxidant in aqueous solution, with the advantage of avoiding the destruction of carbon-carbon double bond, an easy separation of oxidant, and an environmentally benign aspect in a good isolated yield of 80%. The intermediate myrtenal-based 4-methylthiosemicarbazide was prepared by the *N*-acylation reaction of 4-methylthiosemicarbazide with myrtenyl chloride converted from compound **3**. Then, 14 target compounds **6a**–**6n** were synthesized in 72%–95% yields by the one-pot sequential processes involving the cyclization reaction of intermediate **5** under microwave irradiation and the nucleophilic substitution with different alkyl halides.

The structures of the target compounds were characterized by IR, ^1^H-NMR, ^13^C-NMR, and ESI-MS and the related spectra can be found in Supplementary Materials. In IR spectra, the weak absorption bands at about 3045 cm^−1^ were attributed to the stretching vibrations of the unsaturated C–H in the myrtenal moiety. The weak absorption bands at 1604–1640 cm^−1^ and the strong absorption bands at 1456–1522 cm^−1^ were assigned to the vibrations of C=C in the myrtenal moiety and C=N in 1,2,4-triazole moiety, respectively. The absorption bands in the region of 679–710 cm^−1^ were due to the vibrations of C–S–C. In the ^1^H-NMR spectra, the olefinic protons of myrtenal scaffold showed signals at about 6.06 ppm, and the other protons bonded to the saturated carbons of the myrtenal moiety displayed signals in the range of 0.92–2.89 ppm. The characteristic signals at 3.17–3.58 ppm were assigned to the methyl protons of 1,2,4-triazole moiety. The protons on the saturated carbon bonded to the S atom displayed the signals at about 4.10 ppm. The ^13^C-NMR spectra of all the target compounds showed peaks for the olefinic carbons of the myrtenal moiety at 125.35–127.81 ppm and 135.54–135.88 ppm, and the other saturated carbons displayed signals in the region of 21.08–40.33 ppm. For the1,2,4-triazole moiety, the signals at 149.77–151.59 ppm and 155.37–155.86 ppm were assigned to the unsaturated carbons, and at 31.55–32.07 ppm to methyl. The saturated carbon bonded to the S atom displayed the signals at 44.63–44.68 ppm. Their molecular weights were confirmed by the ESI-MS.

### 2.2. Antifungal Activity

The antifungal activities of the target compounds **6a**–**6n** were evaluated by in vitro method against Fusarium wilt on cucumber (*Fusarium oxysporum* f. sp. *cucumerinum*), apple root spot (*Physalospora piricola*), tomato early blight (*Alternaria solani*), speckle on peanut (*Cercospora arachidicola*), and wheat scab (*Gibberella zeae*) at 50 µg/mL. The results are listed in Table 1.

It was found that, at 50 µg/mL, the target compounds presented obviously different antifungal activity against the five tested fungi. Compared with that of the commercial fungicide azoxystrobin (positive control), some compounds exhibited significant inhibitory effect against *P. piricola*, in which compounds **6c** (R = *i*-Pr), **6l** (R = *o*-NO_2_ Bn), and **6a** (R = Et) had inhibitory rates of 98.2%, 96.4%, and 90.7%, respectively, displaying better or comparable antifungal activity than that of the positive control with an inhibition rate of 96.0%. Besides, several compounds displayed moderate activity against *G. zeae*. For example, compounds **6f** (*o*-Me Bn), **6j** (*m*-Cl Bn), and **6i** (*o*-Cl Bn) held 60.2, 56.7, and 50.0% inhibitory rates, respectively. However, the title compounds showed weak activity against the other fungi. It was also found that most of the target compounds showed enhanced activities than that of myrtenal, indicating that the incorporation of 1,2,4-triazole-thioether moiety into the myrtenal molecule was beneficial to the increase of antifungal activity. To our surprise, some compounds showed the difference of activity up to two- to three-fold even in the different small R groups, meaning that the contributory effect on different activity is not only R groups. Even though no reasonable explanation has been found so far, the difference may inspire further investigations.

## 3. Experimental Section

### 3.1. General

The GC analysis was conducted on Agilent 6890 GC (Agilent Technologies Inc., Santa Clara, CA, USA) equipped with column HP-1 (30 m, 0.530 mm, 0.88 µm) and FID. IR spectra were recorded on Nicolet iS50 FT-IR spectrometer (Thermo Scientific Co., Ltd., Madison, WI, USA) (KBr pellet method). NMR spectra were recorded in CDCl_3_ solvent on Bruker Avance III HD 600 MHz spectrometer (Bruker Co., Ltd., Zurich, Switzerland) and chemical shifts are expressed in ppm (δ) downfield relative to TMS as an internal standard. MS spectra were obtained by means of the electrospray ionization (ESI) method on TSQ Quantum Access MAX HPLC-MS instrument (Thermo Scientific Co., Ltd., Waltham, MA, USA). The UV spectra were measured on a Shimadzu UV-1800 spectrophotometer (Shimadzu Corp., Kyoto, Japan). Melting points were determined on a MP420 automatic melting point apparatus (Hanon Instruments Co., Ltd., Jinan, China) and were not corrected. Microwave irradiation-assisted synthesis was carried out on an XO-SM50 ultrasonic microwave reaction system (Nanjing Xianou Instrument Manufacturing Co., Ltd., Nanjing, China). α-Pinene (GC purity 98%) was provided by Wuzhou Pine Chemicals Co., Ltd., Wuzhou, Guangxi, China. Other reagents were purchased from commercial suppliers and used as received.

### 3.2. Synthesis of Myrtenal from α-Pinene

A solution of SeO_2_ (20.0 g, 0.18 mol) in anhydrous EtOH (41.6 mL, 0.71 mol) was refluxed for 10 min, and then the excessive ethanol was removed by distillation to give a colorless transparent liquid, which was dissolved in 1,4-dioxane (10.0 mL). The resulting solution was added slowly to a mechanically stirred mixture of α-pinene (26.8 g, 0.20 mol) and 1,4-dioxane (20 mL) at 60.0 °C, and the reaction mixture was continuously heated to separate the produced mixture of ethanol and water from the reaction system by distillation at 95.0 °C. When the fraction did not produce, the reaction mixture was cooled to room temperature and filtrated to recover metallic selenium. The resulting filtrate was distilled to remove low boiling components, and steam distillation was followed in the presence of sodium sulfite (1.0 g). The distillate was extracted several times with ethyl ether. The separated organic phase was dried over anhydrous sodium sulfate, distilled in a warm bath to remove the ethyl ether, and later fractionally distilled in vacuum to provide myrtenal as a colorless transparent liquid (GC purity 97.2%). Yield 70%, b.p. 81.0–81.5 °C at 0.67 kPa. UV-vis (EtOH) λ_max_ (log ε): 245.0 (3.94) nm; IR (KBr, cm^−1^): 3027 (w, C=C–H), 2808 (w), 2713 (w, O=C–H), 1680 (s, C=O), 1620 (m, C=C); ^1^H-NMR (600 MHz, CDCl_3_): δ = 9.41 (s, 1H), 6.69 (s, 1H), 2.84 (t, 1H), 2.45–2.59 (m, 3H), 2.16 (s, 1H), 1.31 (s, 3H), 1.02 (d, 1H, *J* = 9.2 Hz), 0.72 (s, 3H); ^13^C-NMR (150 MHz, CDCl_3_): δ = 191.2, 154.5, 147.8, 40.9, 38.1, 37.6, 33.0, 31.1, 25.6, 20.9. The IR characteristic absorptions was in agreement with the ones described in the previous reports [12]. ^13^C-NMR spectrum was identical to the one described in the previous report [29], and the assignment of ^1^H-NMR spectrum was confirmed by HMQC data.

### 3.3. Synthesis of Myrtenic Acid 

A solution of NaClO_2_ (8.0 g, 70.0 mmol) in H_2_O (70 mL) was added slowly in 2 h to a stirred mixture of myrtenal (GC purity 97.2%, 7.7 g, 50.0 mmol,) in CH_3_CN (50 mL), NaH_2_PO_4_ (1.6 g) in water (20 mL), H_2_O_2_ (35.0%, 5.0 mL, 52.0 mmol) and polyethylene glycol (PEG-400, 3.0 g) at 10.0 °C with ice-water cooling. The reaction was conducted for 7 h. Then Na_2_SO_3_ (0.5 g) was added to destroy the unreacted HOCl and H_2_O_2_. The resulting mixture was acidified with 10.0% aqueous HCl to pH = 3.0, and extracted several times with diethyl ether. The separated organic phase was washed with saturated sodium bisulfite and deionized waters, respectively, and then dried over with anhydrous sodium sulfate, afterwards, it was distilled in warm bath to remove the ethyl ether, and later distilled in vacuum to provide 7.0 g of myrtenic acid as a colorless transparent viscous liquid, which was placed at room temperature to afford a colorless waxy solid. Yield 80.0%, b.p. 130.0–131.0 °C at 0.53 kPa. UV-vis (EtOH) λ_max_ (log ε): 232.6 (3.98) nm; IR (KBr, cm^−1^): 3500–2500 (s, br, O-H), 1682 (s, C=O), 1623 (m, C=C), 1423 (m, δ C-O), 1273 (m, ν C-O); ^1^H-NMR (600 MHz, CDCl_3_): δ = 10.30 (br, 1H), 6.98 (s, 1H), 2.78 (t, 1H, *J* = 5.6, *J* = 5.5), 2.48–2.39 (m, 3H), 2.13 (m, 1H), 1.33 (s, 3H), 1.11 (d, 1H, *J* = 9.2 Hz), 0.79 (s, 3H); ^13^C-NMR (150 MHz, CDCl_3_): δ = 171.7, 139.7, 139.5, 40.9, 40.2, 37.7, 32.4, 31.3, 25.8, 20.9; ESI-MS *m*/*z*: 164.82[M − H]^−^. The peak at 10.30 ppm in ^1^H-NMR spectrum disappeared when the sample was treated with D_2_O.

### 3.4. Synthesis of Myrtenyl Chloride

Under anhydrous atmosphere, a solution of SOCl_2_ (8.2 g, 99.0%, 68.9 mmol) in benzene (10 mL) was added slowly to a solution of myrtenic acid (10.0 g, 57.4 mmol) in benzene (30 mL) at room temperature. Subsequently, three drops of DMF were added, and the mixture was refluxed for 5 h. Afterwards, the reaction solution was distilled at atmospheric pressure to remove the low boiling components, and in vacuum to give myrtenyl chloride as a faint yellow liquid (GC purity 96.9% determined in methanol). Yield 75.0%, b.p. 76.0–77.0 °C at 0.3 kPa). UV-vis (cyclohexane) λ_max_ (log ε): 250.2 (3.92) nm; IR (KBr, cm^−1^): 1747 (s, C=O), 1616 (m, C=C), 807 (m, C–Cl); ^1^H-NMR (600 MHz, CDCl_3_): δ = 7.29 (s, 1H), 2.85 (m, 1H), 2.48–2.61 (m, 3H), 2.16 (m, 1H), 1.33 (s, 3H), 1.11 (d, 1H, *J* = 9.4 Hz), 0.77 (s, 3H); ^13^C-NMR (150 MHz, CDCl_3_): δ = 166.0, 147.7, 144.5, 42.4, 39.9, 37.9, 33.0, 31.2, 25.6, 20.8.

### 3.5. Synthesis of Myrtenal-Based 4-Methylhydrazinecarbothioamide

Under anhydrous atmosphere, myrtenyl chloride (**4**, 5.1 g, 27.4 mmol) was added slowly to a stirred mixture of *N*-methylhydrazinecarbothioamide (3.0 g, 28.6 mmol), methylene dichloride (40 mL), and five drops of triethylamine in an ice-water bath. When the addition was completed, the mixture was then stirred for 30 min, and for 15 min at room temperature. After quenching with HCl (20.0 mL, 10.0%), the reaction mixture was filtrated. The resulting filter cake was then washed three times with ethyl acetate, and dried under vacuum to provide intermediate **5** as a colorless solid. Yield 86.0%, m.p. 196.1–196.6 °C. UV-vis (EtOH) λ_max_ (log ε): 243.8 (4.21) nm; IR (KBr, cm^−1^): 3276, 3194 (s, N–H), 1664 (s, C=O), 1621 (w, amide II), 1556 (s, N–C=S I), 1244 (s, N–C=S II), 1068 (m, N–C=S III); ^1^H-NMR (600 MHz, CDCl_3_): δ = 9.67 (s, 1H), 9.08 (s, 1H), 7.77 (s, 1H), 6.54 (m, 1H), 2.87 (d, 3H, *J* = 4.3 Hz), 2.67 (t, 1H, *J* = 5.2 Hz), 2.30–2.44 (m, 3H), 2.09 (m, 1H), 1.30 (s, 3H), 1.07 (d, 1H, *J* = 8.9 Hz), 0.77 (s, 3H); ^13^C-NMR (150 MHz, CDCl_3_): δ = 182.9, 166.6, 141.7, 130.7, 41.4, 40.5, 37.6, 31.9, 31.4, 31.4, 26.3, 21.4; ESI-MS *m*/*z*: 253.96 [M + H]^+^.

### 3.6. General Procedure for the One-Pot Sequential Synthesis of Myrtenal-Based 4-Methyl-1,2,4-triazole-thioethers *(**6a**–**6n**)*

Compound **5** (3.9 mmol) and potassium hydroxide (0.5 g) were mixed in anhydrous ethanol (20 mL). The mixture was placed in a program-controlled ultrasonic microwave reaction system, and irradiated under stirring at microwave power 200 W and temperature 80 °C for 1 h. Alkyl halide (4.0 mmol) was then slowly added. After the addition, the mixture was sequentially refluxed for 15 min, and distilled to remove the solvent. The residue was acidified with HCl (20 mL, 10.0%) to pH = 1, and filtrated. The resulting filtrate was basified to pH = 12, and extracted three times with cyclohexane. The resulting organic phase was dried over anhydrous magnesium sulfate, and distilled in vacuo to remove the solvent to provide the target compounds **6a**–**6n**.

*Myrtenal-based 4-methyl-1,2,4-triazole-ethylthioether* (**6a**). Colorless solid. Yield: 78.0%, melting point: 69.3–70.3 °C. UV-vis (cyclohexane) λ_max_ (log ε): 266.2 (3.98) nm; IR (KBr, cm^−1^): 3070 (w, = C–H), 1631 (w, C=C), 1484 (m), 1463 (s, C=N), 708 (m, C–S–C); ^1^H-NMR (600 MHz, CDCl_3_): δ = 6.10 (m, 1H), 3.55 (s, 3H), 3.23–3.19 (q, 2H, *J* = 7.28 Hz), 2.87 (t, 1H, *J* = 5.6 Hz), 2.46–2.57 (m, 3H), 2.21 (m, 1H), 1.41 (t, 3H, *J* = 7.4 Hz), 1.36 (s, 3H), 1.35 (d, 1H, *J* = 9.3 Hz), 0.95 (s, 3H); ^13^C-NMR (150 MHz, CDCl_3_): δ = 155.5, 151.3, 135.8, 127.4, 44.7, 40.3, 37.9, 32.2, 31.9, 31.6, 27.8, 26.0, 21.1, 15.0; ESI-MS *m*/*z*: 264.01 [M + H]^+^.

*Myrtenal-based 4-methyl-1,2,4-triazole-n-propylthioether* (**6b**). Colorless solid. Yield: 77.0%, melting point: 78.8–81.3 °C. UV-vis (cyclohexane) λ_max_ (log ε): 266.0 (4.13) nm;IR (KBr, cm^−1^): 3067 (w, = C–H), 1624 (w, C=C), 1483 (m), 1464 (s, C=N), 706 (m, C–S–C); ^1^H-NMR (600 MHz, CDCl_3_): δ = 6.10 (m, 1H), 3.56 (s, 3H), 3.18 (t, 2H, *J* = 7.2 Hz), 2.86 (t, 1H, *J* = 5.6 Hz), 2.45–2.57 (m, 3H), 2.21 (m, 1H), 1.81–1.76 (m, 2H), 1.36 (s, 3H), 1.35 (d, 1H, *J* = 9.3 Hz), 1.03 (t, 3H, *J* = 7.4 Hz), 0.95 (s, 3H); ^13^C-NMR (150 MHz, CDCl_3_): δ = 155.5, 151.5, 135.7, 127.3, 44.6, 40.3, 37.9, 35.3, 32.2, 31.9, 31.5, 26.0, 23.0, 21.1, 13.2; ESI-MS *m*/*z*: 278.01 [M + H]^+^.

*Myrtenal-based 4-methyl-1,2,4-triazole-i-propylthioether* (**6c**). Pale yellow solid. Yield: 72.0%, melting point: 78.2–79.6 °C. UV-vis (cyclohexane) λ_max_ (log ε): 265.8 (4.04) nm; IR (KBr, cm^−1^): 3053 (w, = C–H), 1635 (w, C=C), 1487 (m), 1461 (s, C=N), 706 (m, C–S–C); ^1^H-NMR (600 MHz, CDCl_3_): δ = 6.12 (m, 1H), 3.77 (m, 1H), 3.58 (s, 3H), 2.89 (t, 1H, *J* = 5.6 Hz), 2.46–2.57 (m, 3H), 2.21 (m, 1H), 1.40 (d, 6H, *J* = 6.8 Hz), 1.37 (s, 3H), 1.35 (d, 1H, *J* = 9.3 Hz), 0.95 (s, 3H); ^13^C-NMR (150 MHz, CDCl_3_): δ = 155.4, 150.7, 135.9, 127.4, 44.6, 40.3, 39.7, 37.9, 32.2, 32.1, 31.6, 26.0, 23.5, 21.1; ESI-MS *m*/*z*: 278.04 [M + H]^+^.

*Myrtenal-based 4-methyl-1,2,4-triazole-n-butylthioether* (**6d**). Pale yellow solid. Yield: 80.0%, melting point: 62.1–63.2 °C. UV-vis (cyclohexane) λ_max_ (log ε): 266.2 (4.13) nm; IR (KBr, cm^−1^): 3064 (w, = C–H), 1628 (w, C=C), 1482 (m), 1463 (s, C=N), 706 (m, C–S–C); ^1^H-NMR (600 MHz, CDCl_3_): δ = 6.10 (m, 1H), 3.55 (s, 3H), 3.20 (t, 2H, *J* = 7.5 Hz), 2.88 (t, 1H, *J* = 5.6 Hz), 2.45–2.57 (m, 3H), 2.21 (m, 1H), 1.76–1.71 (m, 2H), 1.43–1.48 (m, 2H), 1.36 (s, 3H), 1.35 (d, 1H, *J* = 9.5 Hz), 0.95 (s, 3H), 0.93 (t, 3H, *J* = 7.2 Hz); ^13^C-NMR (150 MHz, CDCl_3_): δ = 155.5, 151.6, 135.8, 127.2, 44.7, 40.3, 37.9, 33.1, 32.2, 31.9, 31.6, 31.5, 26.0, 21.8, 21.1, 13.6; ESI-MS *m*/*z*: 292.02 [M + H]^+^.

*Myrtenal-based 4-methyl-1,2,4-triazole-benzylthioether* (**6e**). Colorless solid. Yield: 86.0%, melting point: 108.5–110.3 °C. UV-vis (cyclohexane) λ_max_ (log ε): 264.8 (4.01) nm; IR (KBr, cm^−1^): 3064 (w), 3034 (w, Ar–H, = C–H), 1640 (w, C=C), 1601 (w), 1455 (m, Ar), 1493 (m), 1474 (s, C=N), 704 (s, C–S–C); ^1^H-NMR (600 MHz, CDCl_3_): δ = 7.25–7.30 (m, 5H), 6.04 (m, 1H), 4.33 (s, 2H), 3.24 (s, 3H), 2.84 (t, 1H, *J* = 5.6 Hz), 2.44–2.57 (m, 3H), 2.21 (m, 1H), 1.37 (s, 3H), 1.34 (d, 1H, *J* = 9.0 Hz), 0.94 (s, 3H); ^13^C-NMR (150 MHz, CDCl_3_): δ = 155.6, 150.4, 137.0, 135.8, 129.1, 128.6, 127.7, 127.5, 44.7, 40.3, 38.9, 37.9, 32.2, 31.7, 31.5, 26.0, 21.1; ESI-MS *m*/*z*: 326.01 [M + H]^+^.

*Myrtenal-based 4-methyl-1,2,4-triazole-o-methylbenzylthioether* (**6f**). Colorless solid. Yield: 90.0%, melting point: 90.2–91.3 °C. UV-vis (cyclohexane) λ_max_ (log ε): 266.0 (4.06) nm; IR (KBr, cm^−1^): 3048 (w), 3024 (w, Ar–H, = C–H), 1633 (w, C=C), 1604 (w), 1449 (m, Ar), 1485 (m), 1465 (s, C=N), 700 (m, C–S–C); ^1^H-NMR (600 MHz, CDCl_3_): δ = 7.15–7.19 (m, 2H), 7.03–7.07 (m, 2H), 6.02 (m, 1H), 4.32 (s, 2H), 3.17 (s, 3H), 2.80 (t, 1H, *J* = 5.6 Hz), 2.43–2.56 (m, 3H), 2.40 (s, 3H), 2.20 (m, 1H), 1.36 (s, 3H), 1.33 (d, 1H, *J* = 9.1 Hz), 0.93 (s, 3H); ^13^C-NMR (150 MHz, CDCl_3_): δ = 155.7, 150.4, 136.9, 135.8, 134.7, 130.6, 130.0, 128.1, 127.6,126.2, 44.7, 40.3, 37.9, 37.2, 32.2, 31.6, 31.5, 26.0, 21.1; ESI-MS *m*/*z*: 340.05 [M + H]^+^.

*Myrtenal-based 4-methyl-1,2,4-triazole-m-methylbenzylthioether* (**6g**). Colorless solid. Yield: 95.0%, melting point: 90.2–91.3 °C. UV-vis (cyclohexane) λ_max_ (log ε): 266.4 (3.98) nm; IR (KBr, cm^−1^): 3090 (w), 3044 (w, Ar–H, = C–H), 1630 (w, C=C), 1608 (w), 1455 (m, Ar), 1484 (m), 1467 (s, C=N), 699 (m, C–S–C); ^1^H-NMR (600 MHz, CDCl_3_): δ = 7.16 (t, 1H, *J* = 7.6 Hz), 7.06 (t, 2H, *J* = 7.6 Hz), 7.00 (s, 1H), 6.03 (m, 1H), 4.26 (s, 2H), 3.22 (s, 3H), 2.81 (t, 1H, *J* = 5.6 Hz), 2.43–2.56 (m, 3H), 2.28 (s, 3H), 2.20 (m, 1H), 1.36 (s, 3H), 1.33 (d, 1H, *J* = 9.1 Hz), 0.93 (s, 3H); ^13^C-NMR (150 MHz, CDCl_3_): δ = 155.6, 150.5, 138.3, 136.8, 135.7, 129.7, 128.6, 128.4, 127.6, 126.1, 44.6, 40.3, 39.1, 37.9, 32.2, 31.7, 31.5, 26.0, 21.3; ESI-MS *m*/*z*: 340.05 [M + H]^+^.

*Myrtenal-based 4-methyl-1,2,4-triazole-p-methylbenzylthioether* (**6h**). Colorless solid. Yield: 93.0%, melting point: 104.3–105.4 °C. UV-vis (cyclohexane) λ_max_ (log ε): 215.8 (4.33), 266.6 (4.00) nm; IR (KBr, cm^−1^): 3044 (w), 3017 (w, Ar–H, = C–H), 1636 (w, C=C),1614 (w), 1447 (m, Ar), 1482 (s), 1470 (s, C=N), 708 (m, C–S–C); ^1^H-NMR (600 MHz, CDCl_3_): δ = 7.14 (d, 2H, *J* = 8.0 Hz), 7.07 (d, 2H, *J* = 8.0 Hz), 6.03 (m, 1H), 4.29 (s, 2H), 3.24 (s, 3H), 2.80 (t, 1H, *J* = 5.6 Hz), 2.43–2.56 (m, 3H), 2.31 (s, 3H), 2.21 (m, 1H), 1.36 (s, 3H), 1.33 (d, 1H, *J* = 9.0 Hz), 0.93 (s, 3H); ^13^C-NMR (150 MHz, CDCl_3_): δ = 155.6, 150.6, 137.5, 135.7, 133.8, 129.3, 129.0, 127.6, 44.6, 40.3, 38.5, 37.9, 32.2, 31.7, 31.5, 26.0, 21.1, 21.1; ESI-MS *m*/*z*: 340.02 [M + H]^+^.

*Myrtenal-based 4-methyl-1,2,4-triazole-o-chlorobenzylthioether* (**6i**). Pale yellow solid. Yield: 90.0%, melting point: 104.2–105.4 °C. UV-vis (cyclohexane) λ_max_ (log ε): 265.4 (4.03) nm; IR (KBr, cm^−1^): 3054 (w), 3022 (w, Ar–H, = C–H), 1633 (w, C=C), 1593 (w), 1445 (m, Ar), 1474 (s), 1468 (s) (C=N), 698 (m, C–S–C); ^1^H-NMR (600 MHz, CDCl_3_): δ = 7.37 (d, 1H, *J* = 8.0 Hz), 7.26 (d, 1H, *J* = 7.6 Hz), 7.21 (t, 1H, *J* = 7.6 Hz), 7.12 (t, 1H, *J* = 8.0 Hz), 6.03 (m, 1H), 4.43 (s, 2H), 3.27 (s, 3H), 2.81 (t, 1H, *J* = 5.6 Hz), 2.43–2.56 (m, 3H), 2.40 (s, 3H), 2.20 (m, 1H), 1.36 (s, 3H), 1.32 (d, 1H, *J* = 9.1 Hz), 0.92 (s, 3H); ^13^C-NMR (150 MHz, CDCl_3_): δ = 155.7, 150.2, 135.7, 134.9, 134.2, 131.3, 129.7, 129.2, 127.7, 126.9, 44.6, 40.3, 37.9, 36.5, 32.2, 31.7, 31.5, 26.0, 21.1; ESI-MS *m*/*z*: 359.98 [M + H]^+^.

*Myrtenal-based 4-methyl-1,2,4-triazole-m-chlorobenzylthioether* (**6j**). Colorless solid. Yield: 95.0%, melting point: 81.2–82.2 °C. UV-vis (cyclohexane) λ_max_ (log ε): 264.8 (4.03) nm; IR (KBr, cm^−1^): 3086 (w), 3052 (w, Ar–H, = C–H), 1632 (w, C=C), 1596 (w), 1455.49 (m, Ar), 1475 (s), 1468 (s, C=N), 694 (m, C–S–C); ^1^H-NMR (600 MHz, CDCl_3_): δ = 7.25–7.17 (m, 4H), 6.05 (m, 1H), 4.30 (s, 2H), 3.30 (s, 3H), 2.81 (t, 1H, *J* = 5.6 Hz), 2.44–2.56 (m, 3H), 2.20 (m, 1H), 1.36 (s, 3H), 1.33 (d, 1H, *J* = 9.1 Hz), 0.94 (s, 3H); ^13^C-NMR (150 MHz, CDCl_3_): δ = 155.8, 150.1, 139.1, 135.6, 134.4, 129.9, 129.0, 127.9, 127.3, 44.7, 40.3, 38.0, 37.9, 32.2, 31.7, 31.5, 26.0, 21.1; ESI-MS *m*/*z*: 359.94 [M + H]^+^.

*Myrtenal-based 4-methyl-1,2,4-triazole-p-chlorobenzylthioether* (**6k**). Colorless solid. Yield: 91.0%, melting point: 103.5–104.9 °C. UV-vis (cyclohexane) λ_max_ (log ε): 226.0 (4.35), 264.2 (3.97) nm; IR (KBr, cm^−1^): 3051 (w), 3022 (w, Ar–H, = C–H), 1641 (w, C=C), 1593 (w, Ar), 1488 (s), 1456 (s, C=N), 702 (m, C–S–C); ^1^H-NMR (600 MHz, CDCl_3_): δ = 7.23 (s, 4H), 6.04 (m, 1H), 4.31 (s, 2H), 3.31 (s, 3H), 2.81 (t, 1H, *J* = 5.6 Hz), 2.44–2.56 (m, 3H), 2.20 (m, 1H), 1.36 (s, 3H), 1.33 (d, 1H, *J* = 9.2 Hz), 0.93 (s, 3H); ^13^C-NMR (150 MHz, CDCl_3_): δ = 155.7, 150.2, 135.7, 133.6, 130.5, 128.9, 128.7, 127.7, 44.7, 40.3, 38.0, 37.6, 32.2, 31.7, 31.5, 26.0, 21.1; ESI-MS *m*/*z*: 359.97 [M + H]^+^.

*Myrtenal-based 4-methyl-1,2,4-triazole-o-nitrobenzylthioether* (**6l**). Pale yellow solid. Yield: 91.2%, melting point: 81.2–82.3 °C. UV-vis (EtOH) λ_max_ (log ε): 260.6 (4.09) nm; IR (KBr, cm^−1^): 3066 (w), 3048 (w, Ar–H, = C–H), 1609 (w, C=C), 1579 (w, Ar), 1522 (s), 1469 (m, C=N), 710 (m, C–S–C); ^1^H-NMR (600 MHz, CDCl_3_): δ = 8.07 (d, 1H, *J* = 8.3 Hz), 7.73 (d, 1H, *J* = 7.6 Hz), 7.53 (t, 1H, *J* = 7.6 Hz), 7.44 (t, 1H, *J* = 8.3 Hz), 6.05 (m, 1H), 4.77 (s, 2H), 3.40 (s, 3H), 2.84 (t, 1H, *J* = 5.6 Hz), 2.43–2.55 (m, 3H), 2.20 (m, 1H), 1.36 (s, 3H), 1.32 (d, 1H, *J* = 9.2 Hz), 0.92 (s, 3H); ^13^C-NMR (150 MHz, CDCl_3_): δ = 155.8, 150.6, 147.7, 135.6, 133.7, 133.5, 133.3, 128.9, 127.5, 125.4, 44.7, 40.3, 37.9, 34.7, 32.2, 31.8, 31.5, 26.0, 21.1; ESI-MS *m*/*z*: 370.61 [M + H]^+^.

*Myrtenal-based 4-methyl-1,2,4-triazole-m-nitrobenzylthioether* (**6m**). Faint yellow solid. Yield: 90.5%, melting point: 96.8–97.5 °C. UV-vis (EtOH) λ_max_ (log ε): 262.0 (4.10) nm; IR (KBr, cm^−1^): 3090 (w), 3072 (w, Ar–H, = C–H), 1636 (w, C=C), 1585 (w, Ar), 1522 (s), 1463 (s, C=N), 680 (m, C–S–C); ^1^H-NMR (600 MHz, CDCl_3_): δ = 8.22 (s, 1H), 8.11 (d, 1H, *J* = 8.3Hz), 7.76 (d, 1H, *J* = 7.6 Hz), 7.46 (t, 1H, *J* = 8.0 Hz), 6.06 (m, 1H), 4.49 (s, 2H), 3.42 (s, 3H), 2.82 (t, 1H, *J* = 5.6 Hz), 2.44–2.56 (m, 3H), 2.20 (m, 1H), 1.36 (s, 3H), 1.32 (d, 1H, *J* = 9.3 Hz), 0.93 (s, 3H); ^13^C-NMR (150 MHz, CDCl_3_): δ = 155.8, 149.9, 148.3, 139.4, 135.5, 135.5, 129.5, 127.8, 123.9, 122.7, 44.7, 40.3, 37.9, 36.7, 32.2, 31.8, 31.5, 26.0, 21.1; ESI-MS *m*/*z*: 370.62 [M + H]^+^.

*Myrtenal-based 4-methyl-1,2,4-triazole-p-nitrobenzylthioether* (**6n**). Faint yellow solid. Yield: 89.8%, melting point: 119.9–120.6 °C. UV-vis (EtOH) λ_max_ (log ε): 264.2 (4.18) nm; IR (KBr, cm^−1^): 3072 (w), 3040 (w, Ar–H, = C–H), 1605 (w, C=C), 1597 (w, Ar), 1518 (s), 1490 (m, C=N), 704 (m, C–S–C); ^1^H-NMR (600 MHz, CDCl_3_): δ = 8.13 (d, 2H, *J* = 8.8 Hz), 7.56 (d, *J* = 8.8Hz), 6.06 (m, 1H), 4.48 (s, 2H), 3.40 (s, 3H), 2.82 (t, 1H, *J* = 5.6 Hz), 2.44–2.56 (m, 3H), 2.20 (m, 1H), 1.36 (s, 3H), 1.32 (d, 1H, *J* = 9.1 Hz), 0.92 (s, 3H); ^13^C-NMR (150 MHz, CDCl_3_): δ = 155.9, 149.8, 147.3, 144.9, 135.5, 130.1, 127.8, 123.8, 44.7, 40.3, 37.9, 36.6, 32.2, 31.8, 31.5, 26.0, 21.1; ESI-MS *m*/*z*: 370.62 [M + H]^+^.

### 3.7. Antifungal Activity Test

The tested compound was dissolved in acetone. Sorporl-144 (200 μg/mL) emulsifier was added to dilute the solution to 500 μg/mL. Then, 1 mL solution of the tested compound was poured into a culture plate, and then 9 mL PSA culture medium was added to obtain the flat containing 50 μg/mL tested compound. A bacterium tray of 5-mm diameter cut along the external edge of the mycelium was transferred to the flat containing the tested compound and put in equilateral triangular style in duplicate. Later, the culture plate was cultured at 24 ± 1 °C and the expanded diameter of the bacterium tray was measured after 48 h and compared with that treated with aseptic distilled water to calculate the relative inhibition percentage.

Relative inhibitory rate (%) = (*CK* −*PT*)/*CK* × 100%

where *CK* is the extended diameter of the circle of mycelium during the blank assay and *PT* is the extended diameter of the circle of mycelium during testing.

## 4. Conclusions

Fourteen novel myrtenal-based 4-methyl-1,2,4-triazole-thioethers were designed, synthesized, characterized, and evaluated for their antifungal activity. As a result, at 50 µg/mL, the target compounds exhibited best antifungal activity against *P. piricola*, in which compounds **6c**, **6l**, and **6a** exhibited 98.2%, 96.4%, and 90.7% inhibition rates, respectively, showing better or comparable antifungal activity than that of the commercial fungicide azoxystrobin with the inhibition rate of 96.0%. Thus, these three new analogs can serve as starting points for additional antifungal studies.

## Figures and Tables

**Table 1:** Antifungal activity of the target compounds **6a**–**6n** at 50 µg/mL.

Compounds	Relative Inhibition Rate (%) against the Fungi				
	*F. oxysporum* f. sp. *cucumerinum*	*C. arachidicola*	*P. piricola*	*A. solani*	*G. zeae*
**6a** (R = Et)	30.9	36.7	90.7	28.9	27.6
**6b** (R = *n*-Pr)	35.6	46.7	54.6	47.8	30.6
**6c** (R = *i*-Pr)	37.9	36.7	98.2	28.9	40.9
**6d** (*n*-Bu)	47.2	46.7	36.8	47.8	36.5
**6e** (R = Bn)	20.5	33.3	43.3	38.6	45.6
**6f** (*o*-Me Bn)	20.5	33.3	70.5	32.9	60.2
**6g** (*m*-Me Bn)	23.2	36.7	48.1	47.1	49.0
**6h** (*p*-Me Bn)	30.0	25.4	20.0	18.3	33.3
**6i** (*o*-Cl Bn)	40.0	33.1	30.0	26.7	50.0
**6j** (*m*-Cl Bn)	30.0	40.8	30.0	18.3	56.7
**6k** (*p*-Cl Bn)	20.0	25.4	20.0	18.3	36.7
**6l** (R = *o*-NO_2_ Bn)	37.9	46.7	96.4	45.1	42.4
**6m** (R = *m*-NO_2_ Bn)	58.8	36.7	51.1	50.5	37.9
**6n** (R = *p*-NO_2_ Bn)	30.9	26.7	36.8	42.4	30.6
Myrtenal	25.0	28.2	37.8	41.3	31.7
Azoxystrobin	87.5	92.3	96.0	91.3	90.9

*Azoxystrobin*, a currently commercial fungicide was used as a positive control.

## Supplementary Materials

Supplementary materials can be accessed at: http://www.mdpi.com/1420-3049/22/2/193/s1.
