# Supplementary material for: Synthesis and Antifungal Activity of Novel Myrtenal-Based 4-Methyl-1,2,4-triazole-thioethers
Source: Molecules. 2017 Jan 24;22(2):193. doi: 10.3390/molecules22020193 (PMC6155697; doi:10.3390/molecules22020193)
Supplement: Supplementary file 1 [file molecules-22-00193-s001.pdf]

# Supplementary Materials: Synthesis and Antifungal Activity of Novel Myrtenal-Based 4-Methyl-1,2,4-triazole-thioethers

**Gui-Shan Lin, Wen-Gui Duan, Lin-Xiao Yang, Min Huang and Fu-Hou Lei**

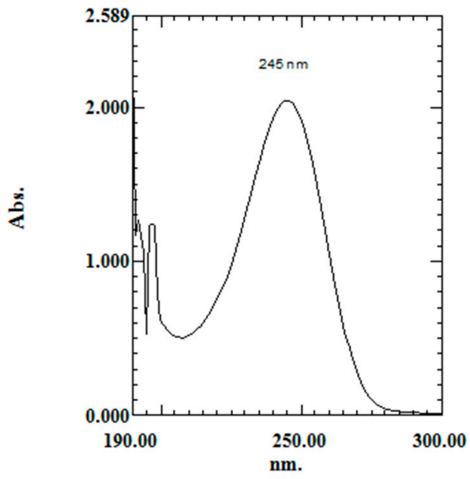


**Figure S1.** UV-vis spectrum of myrtenal (**2**) in EtOH.

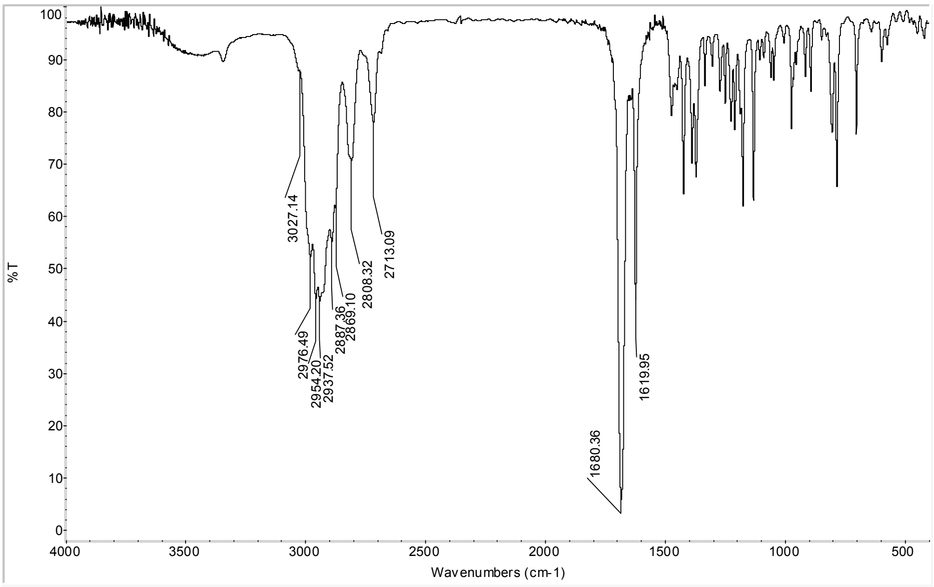


**Figure S2.** FTIR spectrum of myrtenal (**2**).

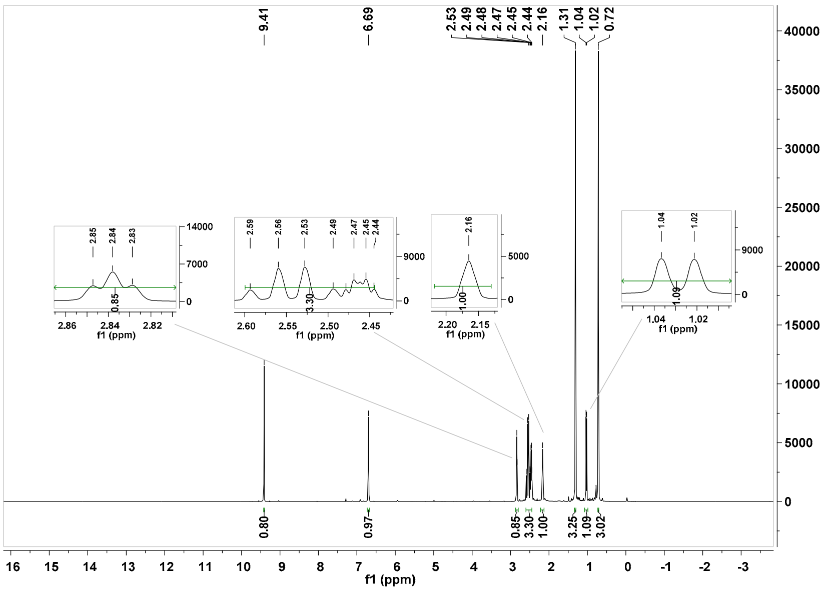


**Figure S3.** $^{1}$H-NMR spectrum of myrtenal (**2**) in $CDCl_3$.

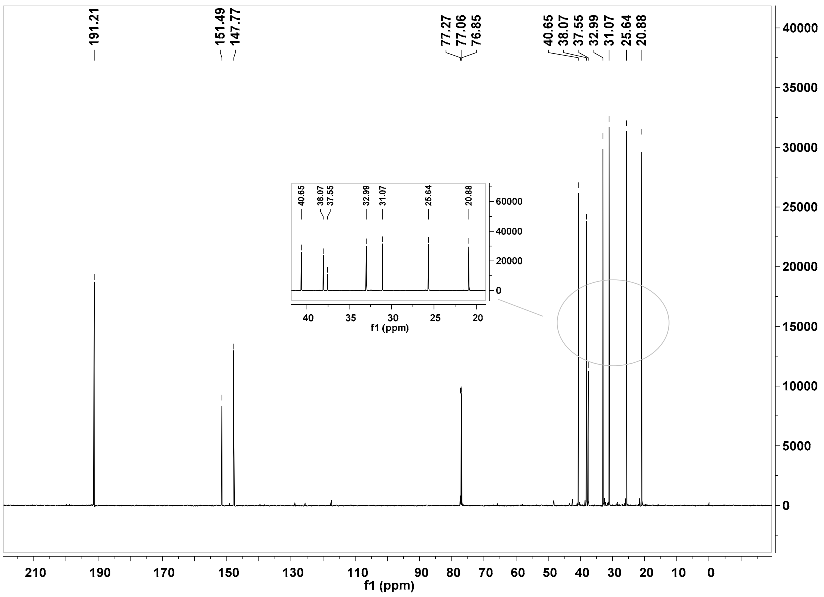


**Figure S4.** $^{13}$C-NMR spectrum of myrtenal (**2**) in $CDCl_3$.

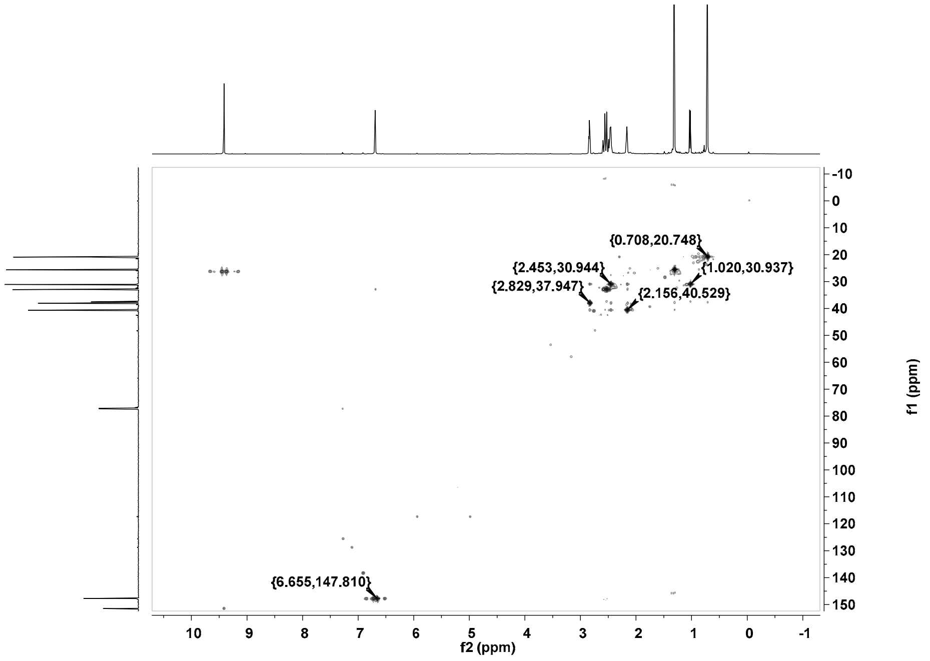


**Figure S5a.** HMQC spectrum of myrtenal (**2**) in $CDCl_3$.

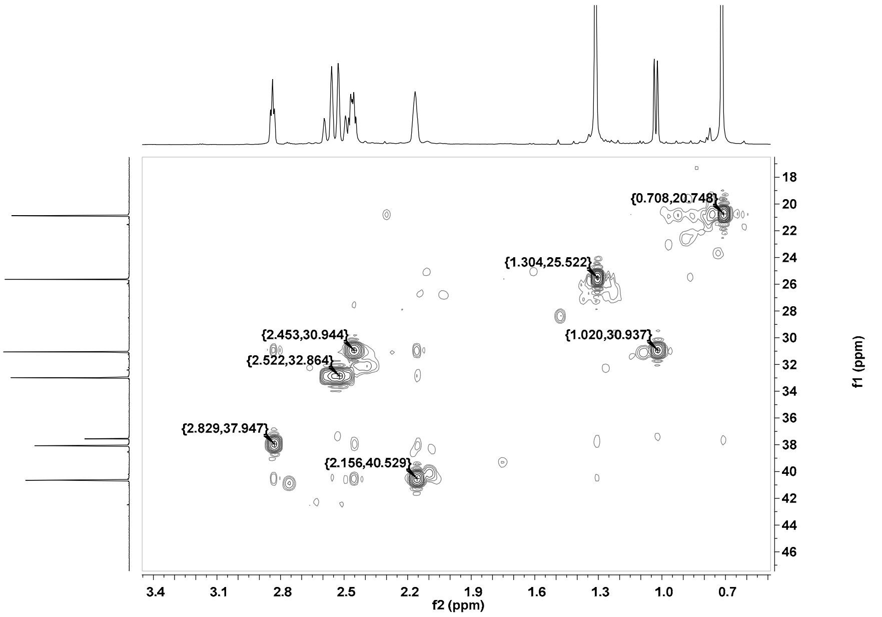


**Figure S5b.** Expanded HMQC spectrum of myrtenal (2) in $CDCl_3$.

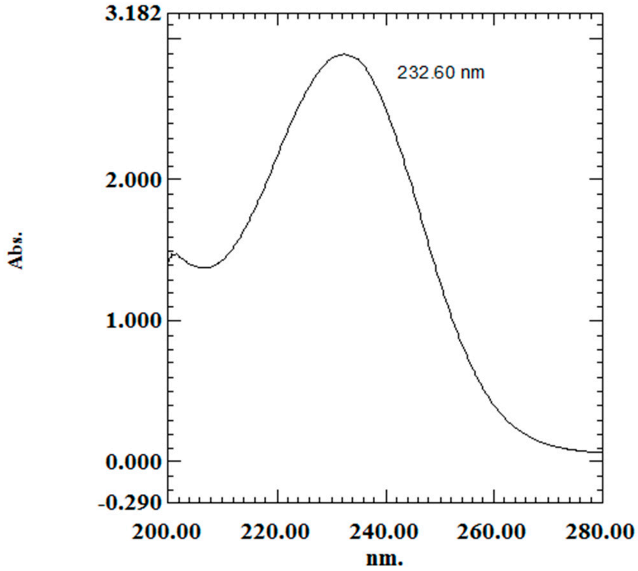


**Figure S6.** UV-vis spectrum of myrtenic acid (**3**) in EtOH.

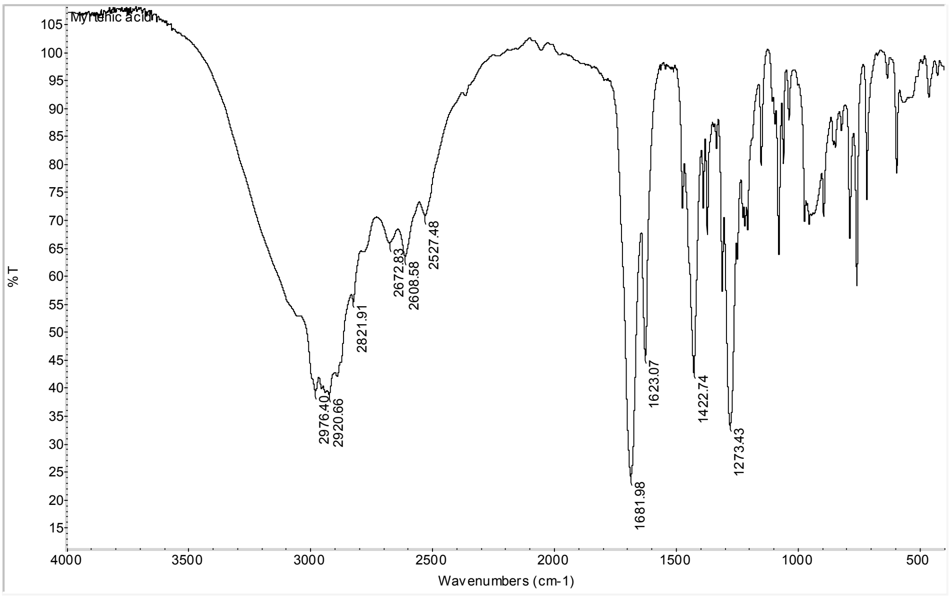


**Figure S7.** FTIR spectrum of myrtenic acid (**3**).

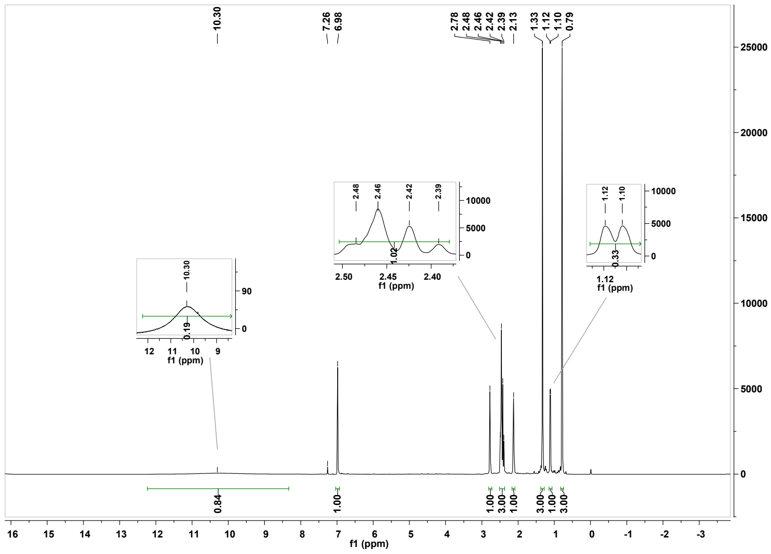


**Figure S8.** $^{1}H$-NMR spectrum of myrtenic acid (**3**) in $CDCl_3$.

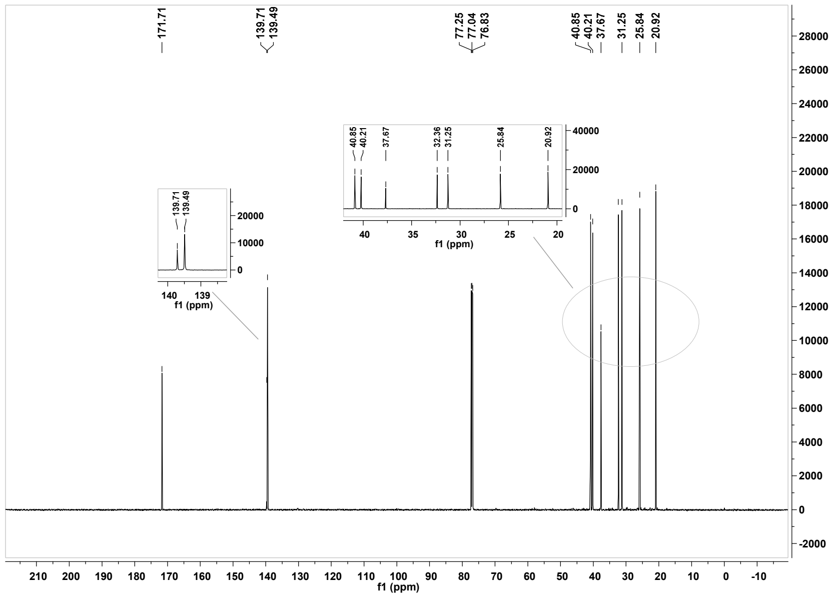


**Figure S9.** $^{13}C$-NMR spectrum of myrtenic acid (**3**) in $CDCl_3$.

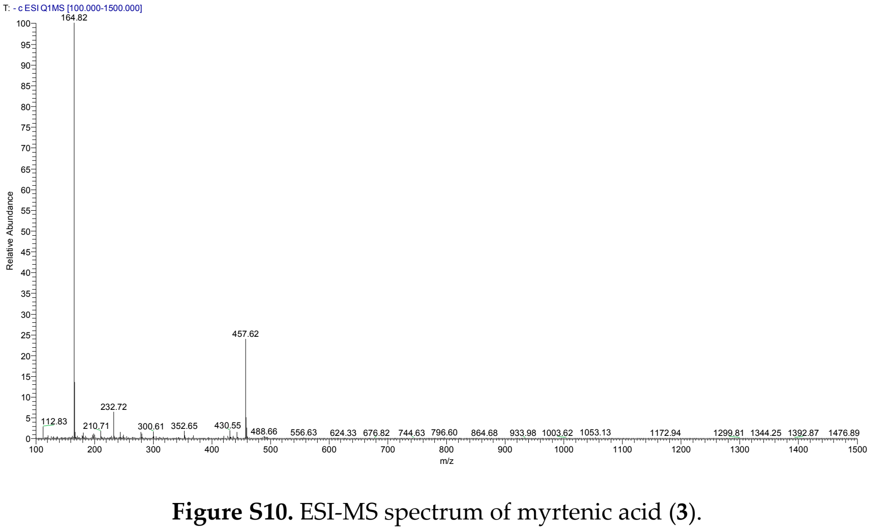


**Figure S10.** ESI-MS spectrum of myrtenic acid (**3**).

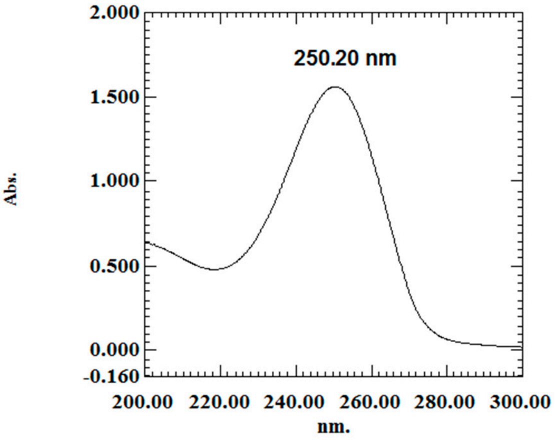


**Figure S11.** UV-vis spectrum of myrtenyl chloride (**4**) in cyclohexane.

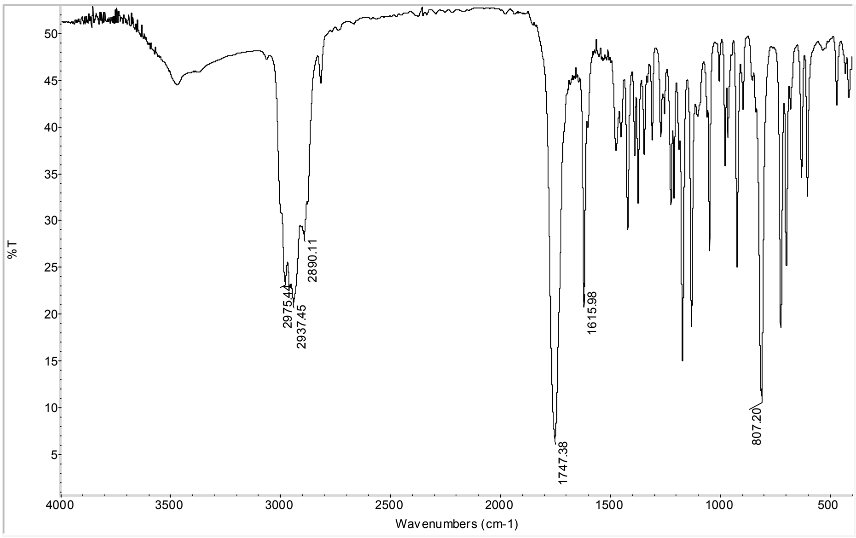


**Figure S12.** FTIR spectrum of myrtenyl chloride (**4**).

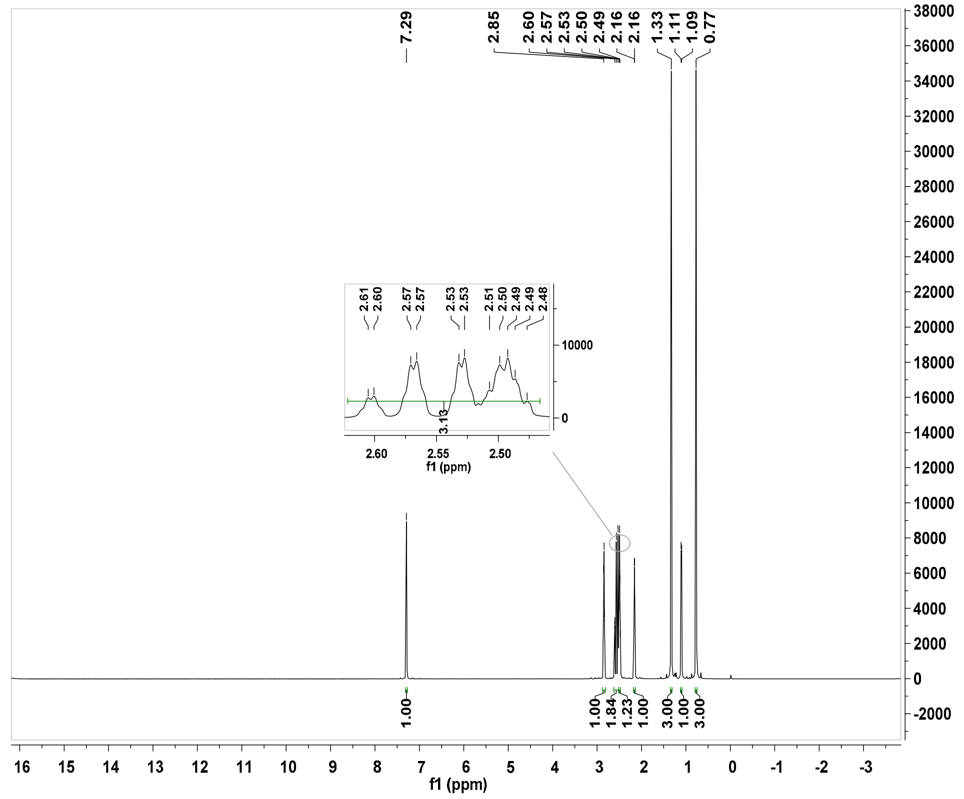


**Figure S13.** $^{1}H$-NMR spectrum of myrtenyl chloride (**4**) in $CDCl_3$.

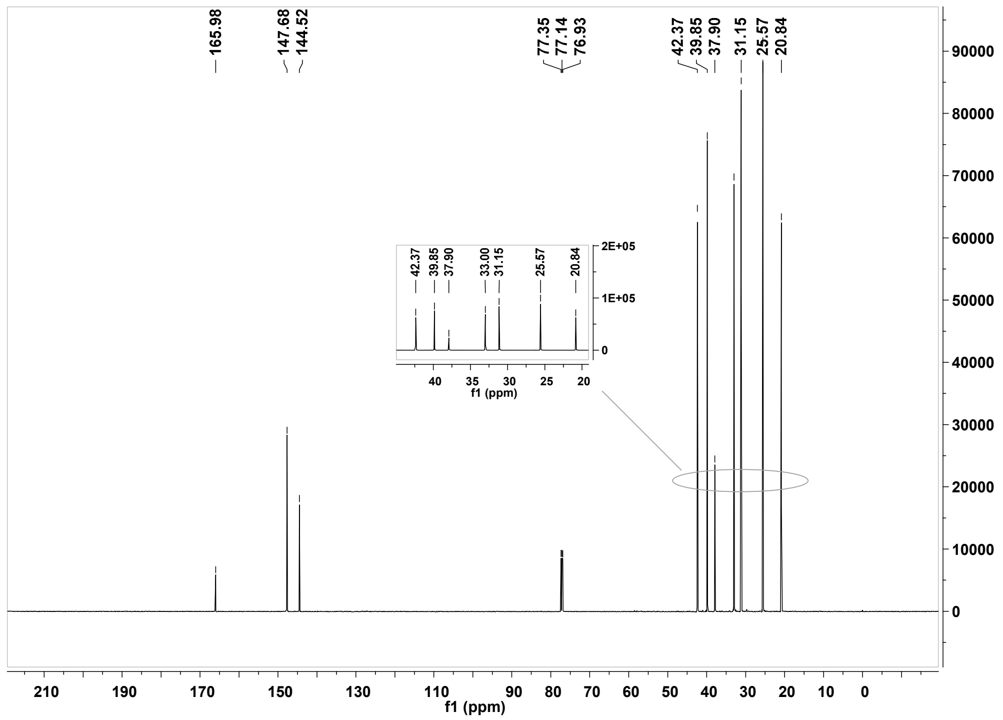


**Figure S14.** $^{13}C$-NMR spectrum of myrtenyl chloride (**4**) in $CDCl_3$.

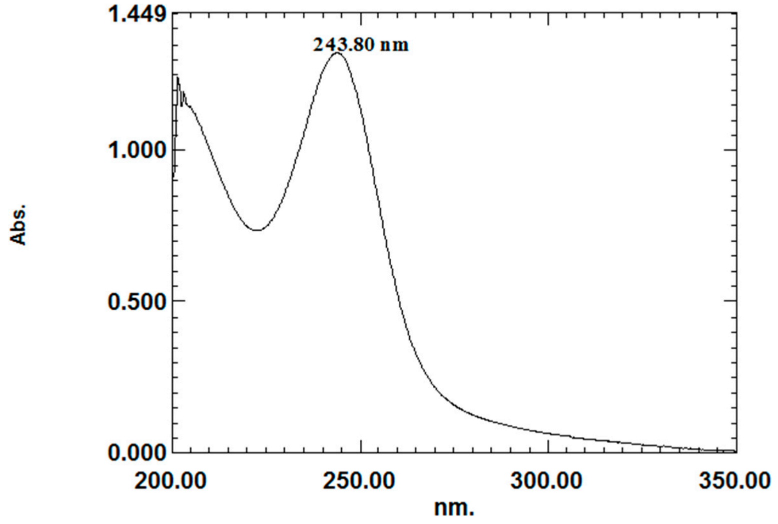


**Figure S15.** UV-vis spectrum of (**5**) in EtOH.

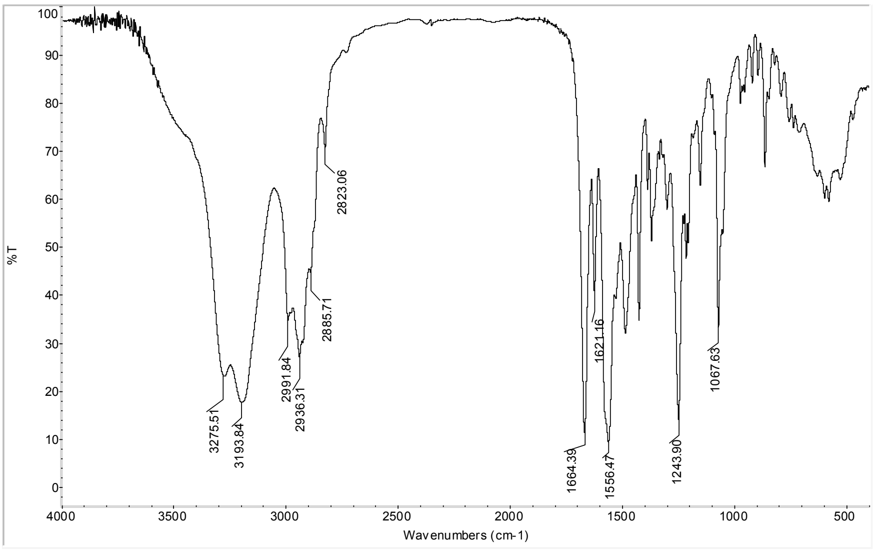


**Figure S16.** FTIR spectrum of 2-(myrtenoic carbonyl)-*N*-methylhydrazinecarbothioamide (**5**).

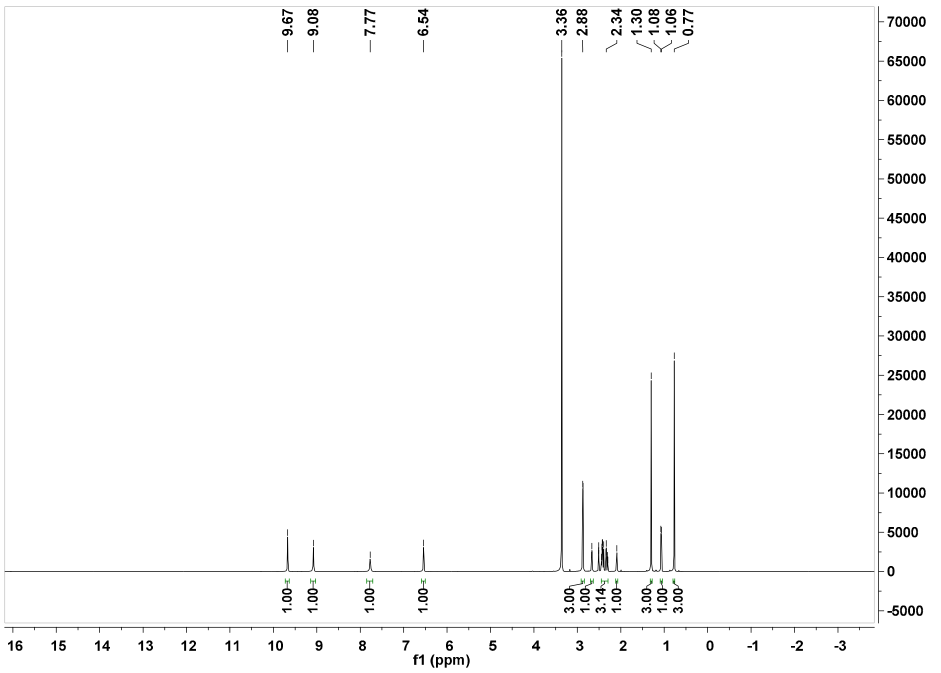


**Figure S17.** $^{1}$H-NMR 2-(myrtenoic carbonyl)-*N*-methylhydrazinecarbothioamide (**5**) in $CDCl_3$.

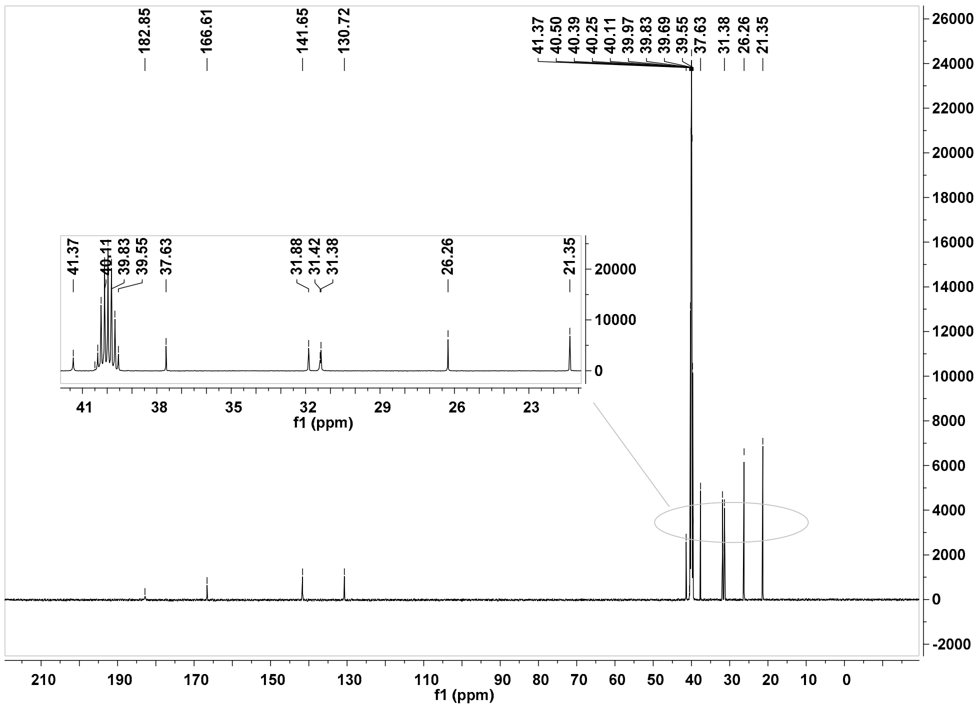


**Figure S18.** $^{13}$C-NMR spectrum of 2-(myrtenoic carbonyl)-*N*-methylhydrazinecarbothioamide (**5**) in $CDCl_3$.

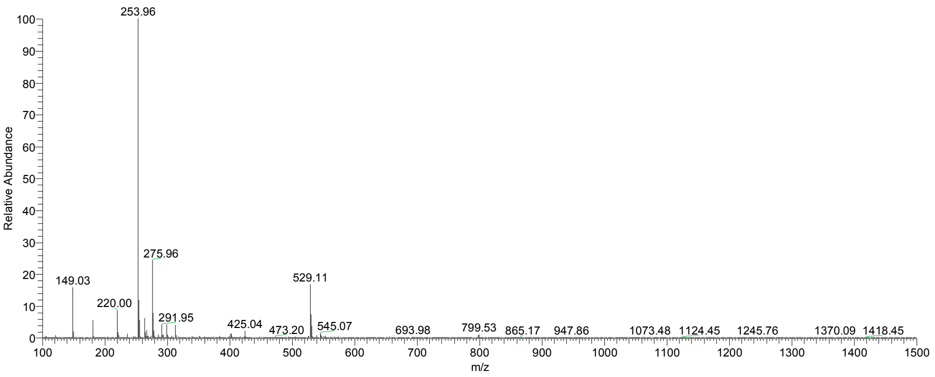


**Figure S19.** ESI-MS spectrum of (**5**).

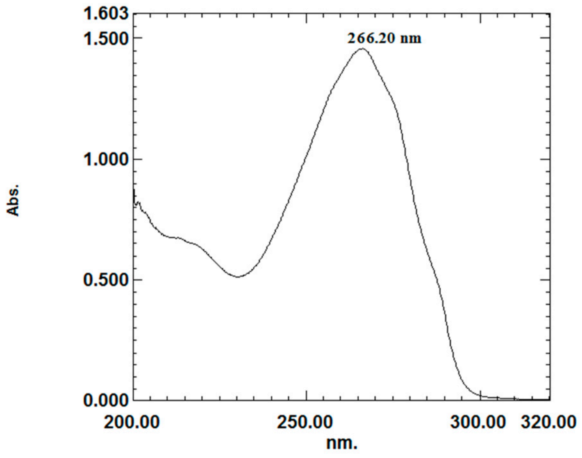


**Figure S20.** UV-vis spectrum of the target compound **6a** in cyclohexane.

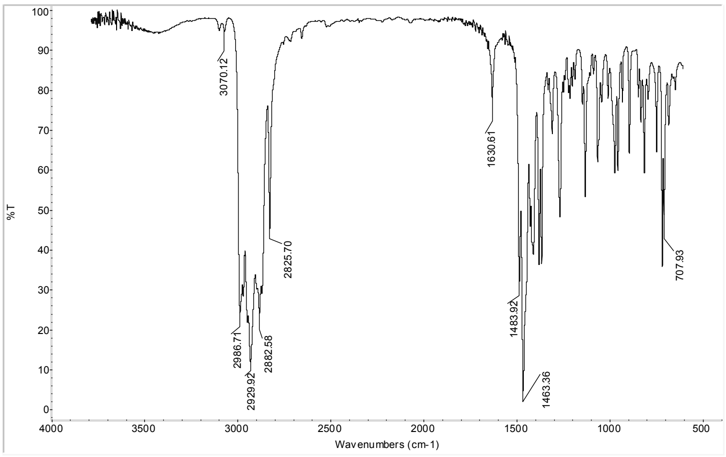


**Figure S21.** FTIR spectrum of the target compound **6a**.

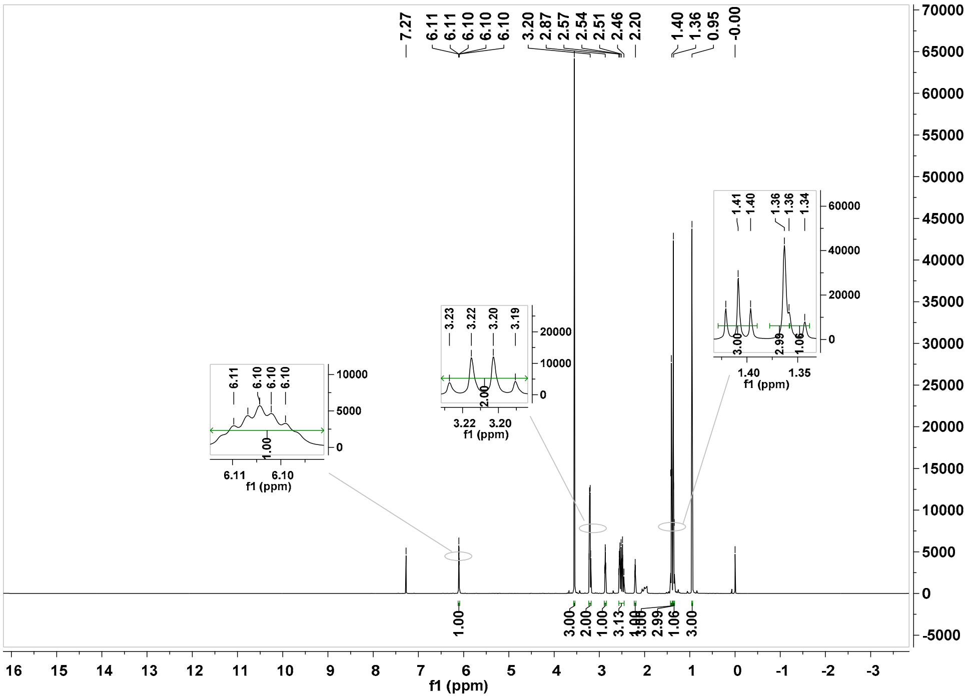


**Figure S22.** $^{1}$H-NMR spectrum of the target compound **6a** in $CDCl_3$.

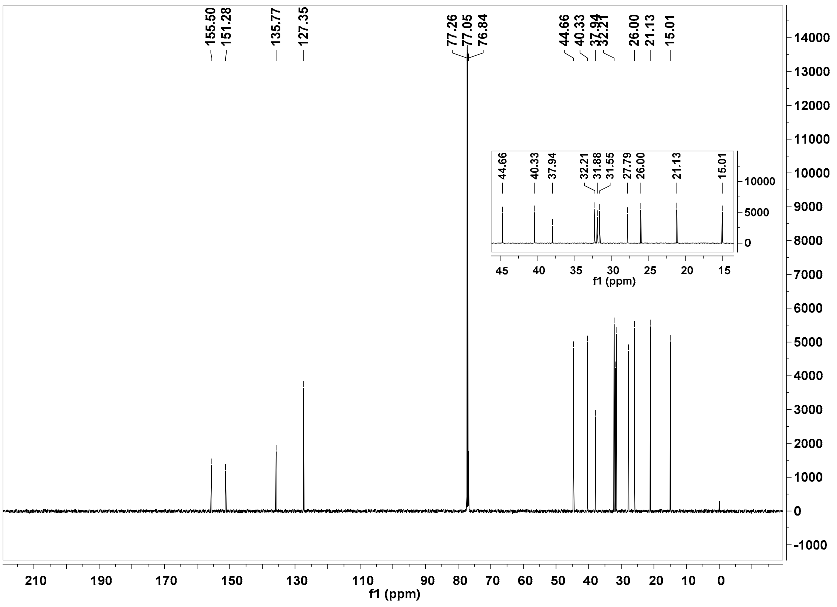


**Figure S23.** $^{13}$C-NMR spectrum of the target compound **6a** in $CDCl_3$.

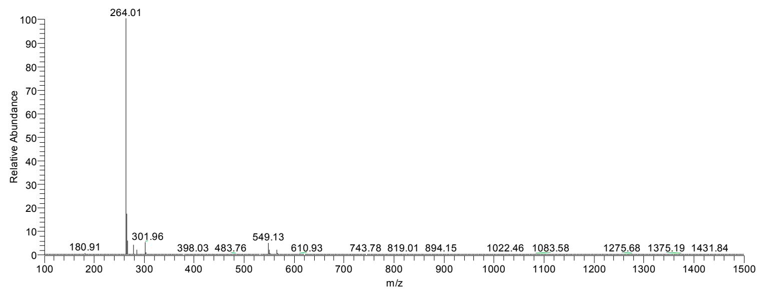

**Figure S24.** ESI-MS spectrum of the target compound **6a**.

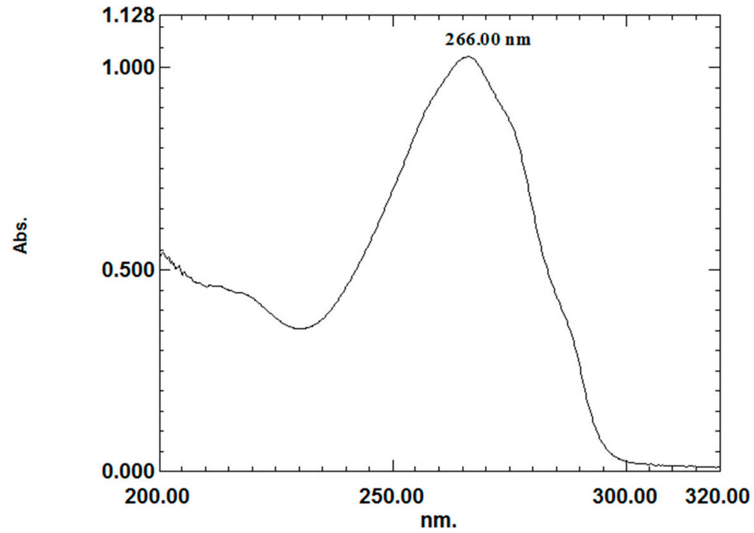


**Figure S25.** UV-vis spectrum of the target compound **6b** in cyclohexane.

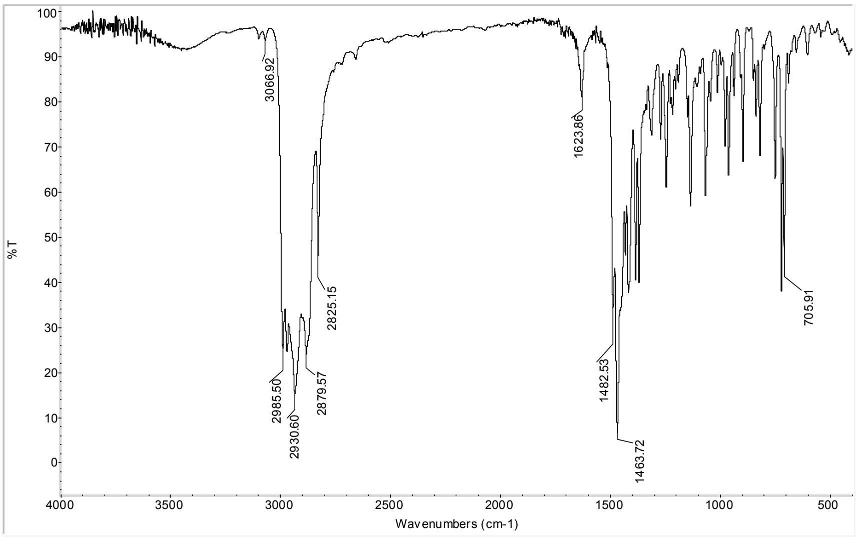


**Figure S26.** FTIR spectrum of the target compound **6b**.

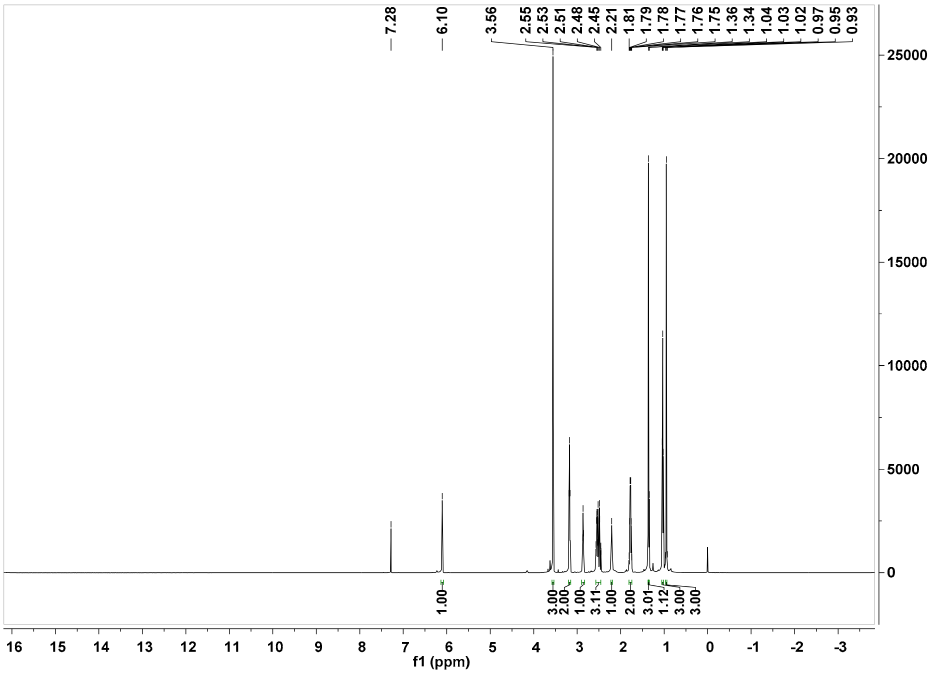


**Figure S27.** $^{1}$H-NMR spectrum of the target compound **6b** in $CDCl_3$.

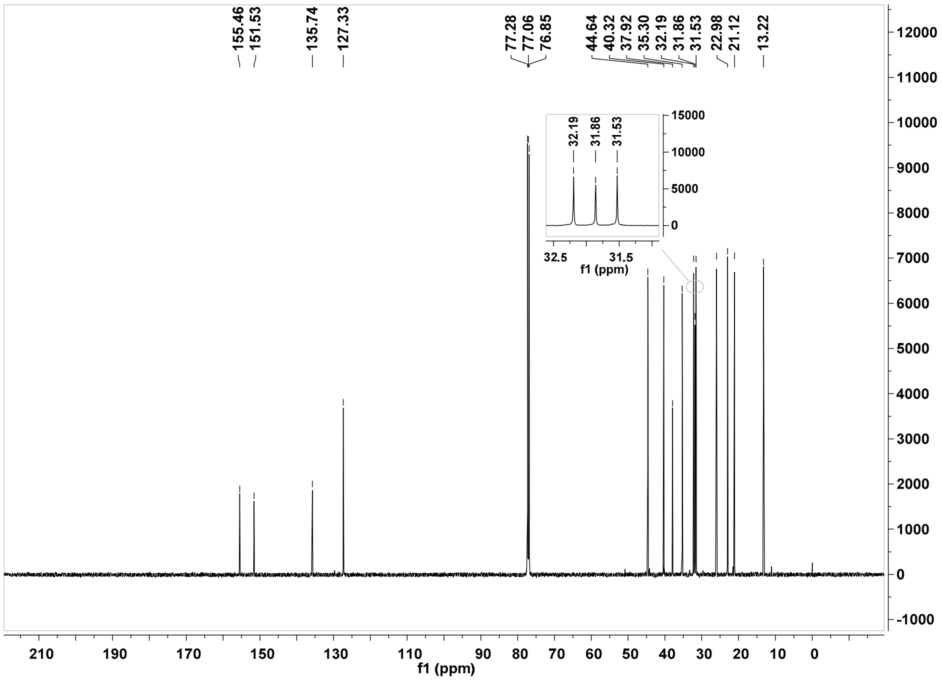


**Figure S28.** $^{13}$C-NMR spectrum of the target compound **6b** in $CDCl_3$.

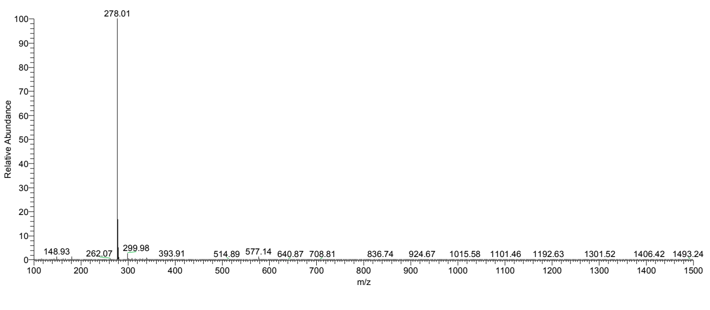

**Figure S29.** ESI-MS spectrum of the target compound **6b**.

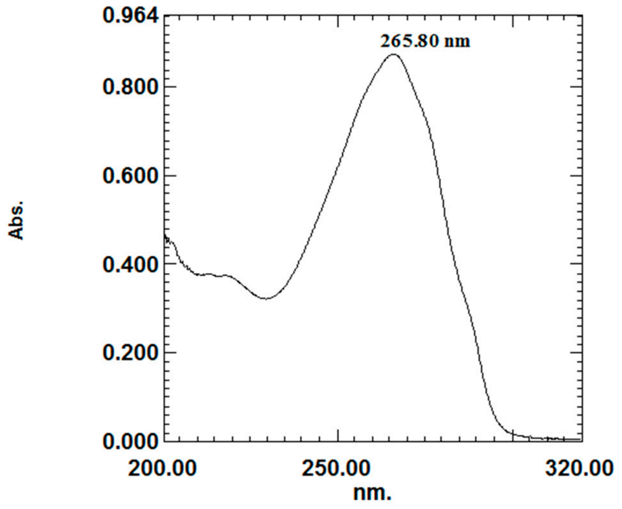


**Figure S30.** UV-vis spectrum of the target compound **6c** in cyclohexane.

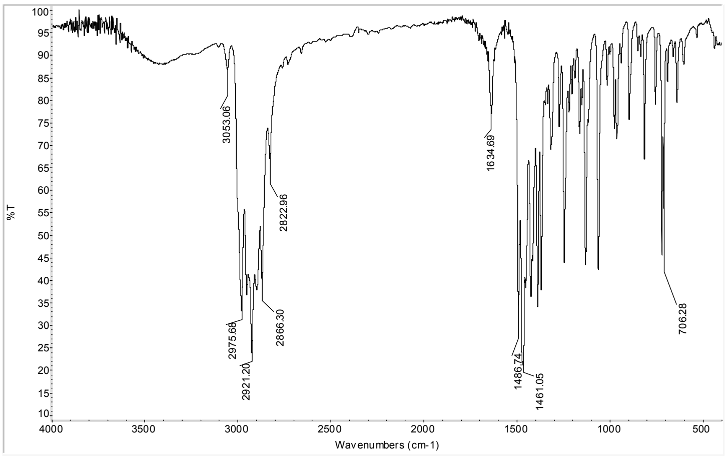


**Figure S31.** FTIR spectru m of the target compound **6c**.

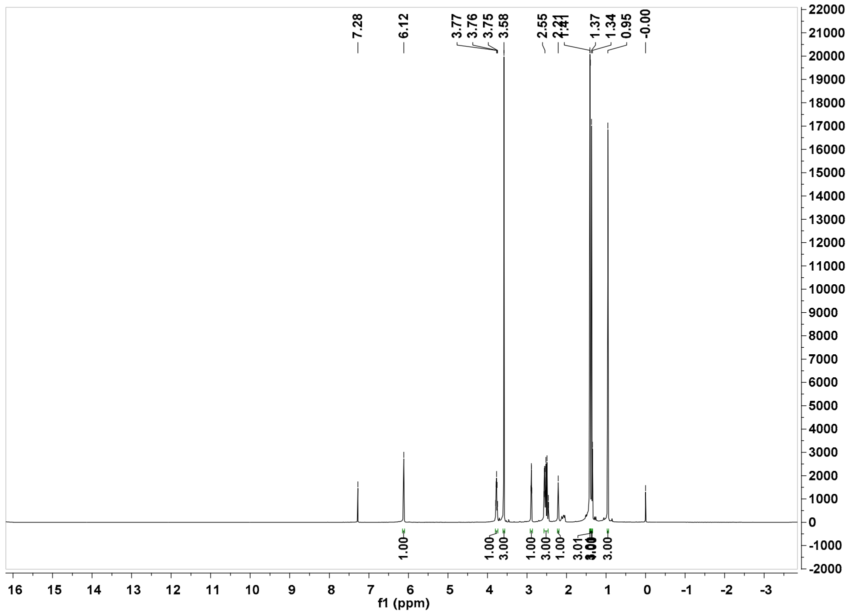


**Figure S32.** $^{1}$H-NMR spectrum of the target compound **6c** in $CDCl_3$.

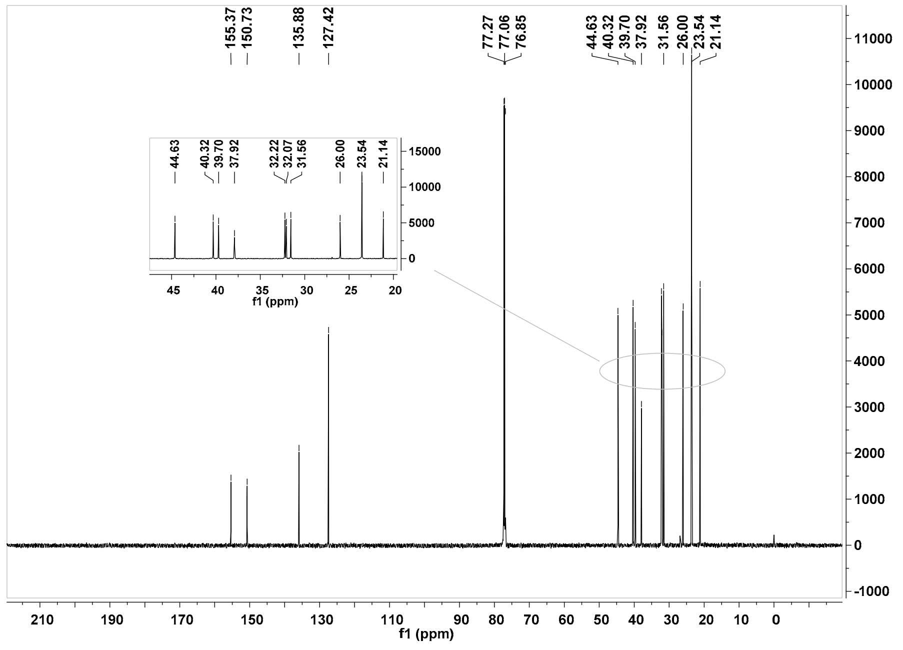


**Figure S33.** $^{13}$C-NMR spectrum of the target compound **6c** in $CDCl_3$.

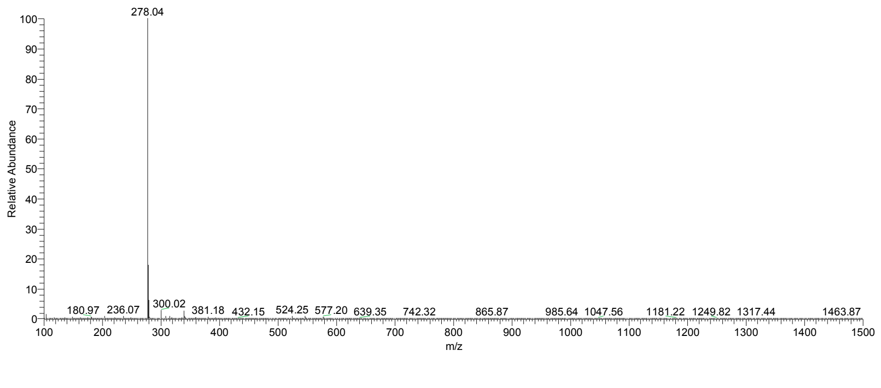

**Figure S34.** ESI-MS spectrum of the target compound **6c**.

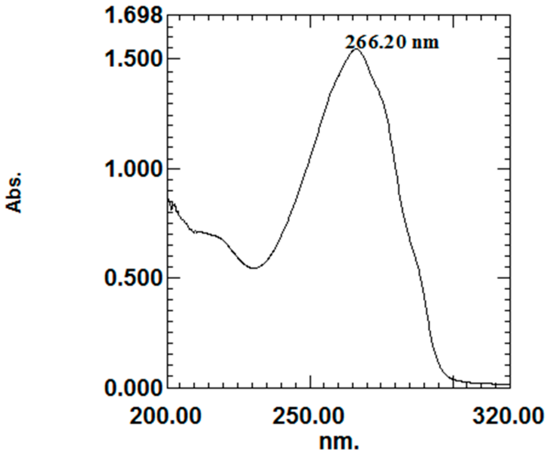


**Figure S35.** UV-vis spectrum of the target compound **6d** in cyclohexane.

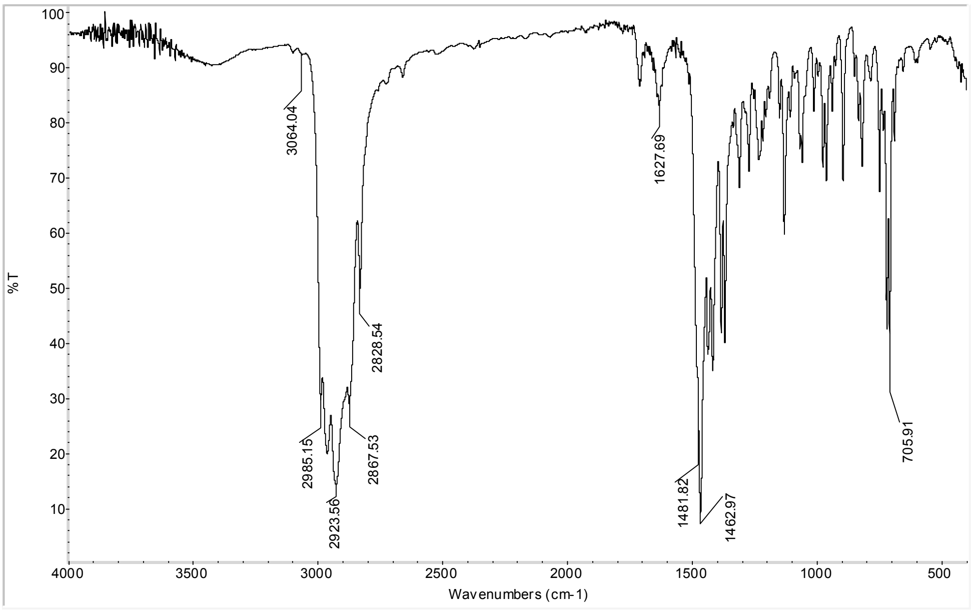


**Figure S36.** FTIR spectrum of the target compound **6d**.

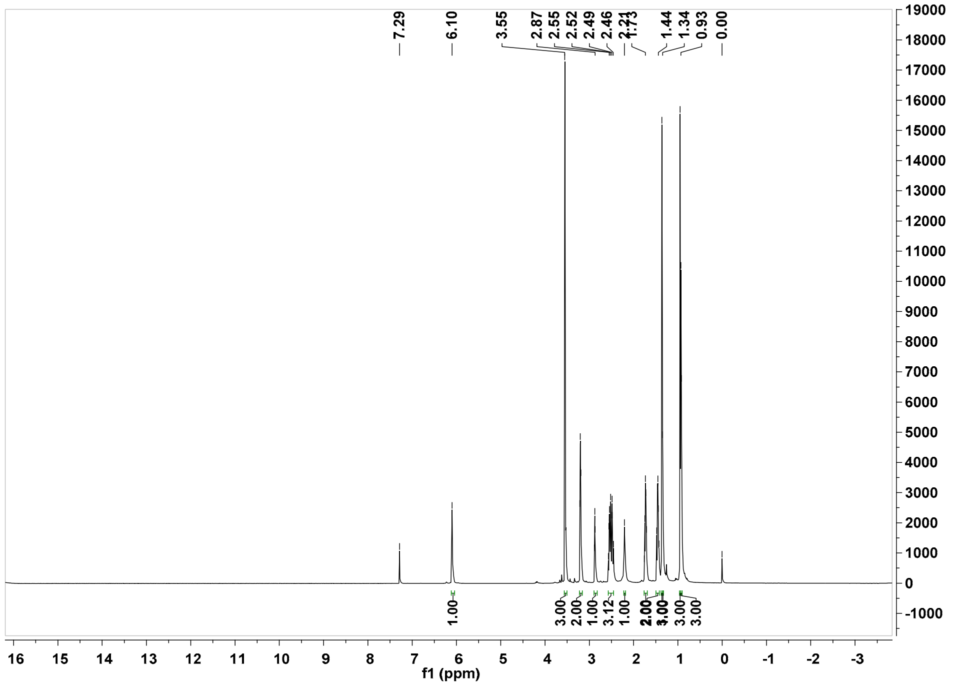


**Figure S37.** $^{1}$H-NMR spectrum of the target compound **6d** in $CDCl_3$.

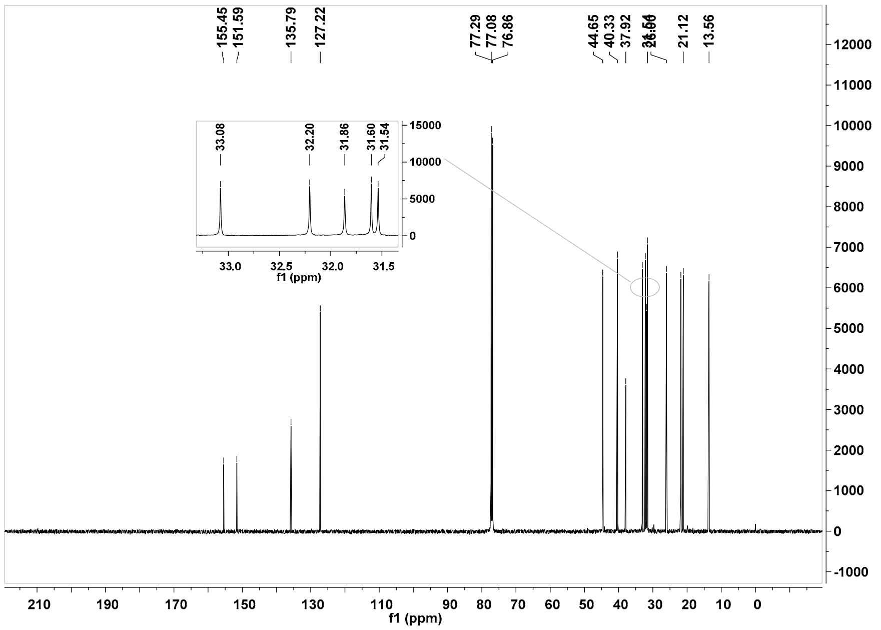


**Figure S38.** $^{13}$C-NMR spectrum of the target compound **6d** in $CDCl_3$.

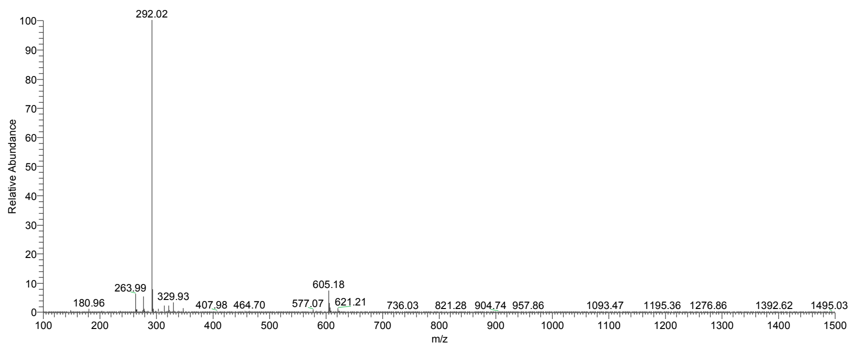

**Figure S39.** ESI-MS spectrum of the target compound **6d**.

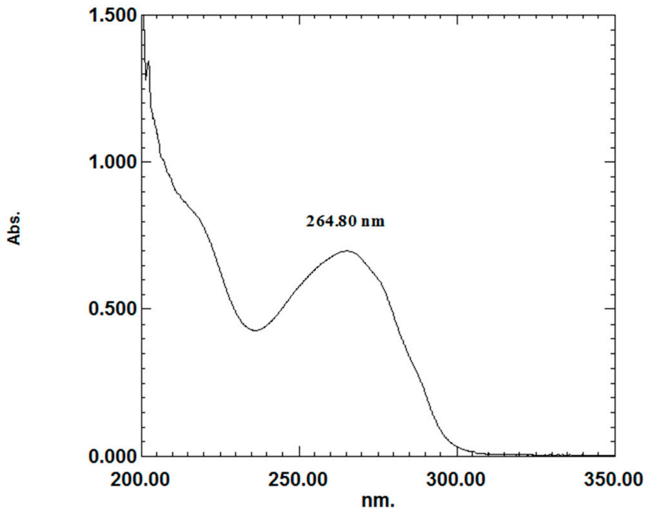


**Figure S40.** UV-vis spectrum of the target compound **6e** in cyclohexane.

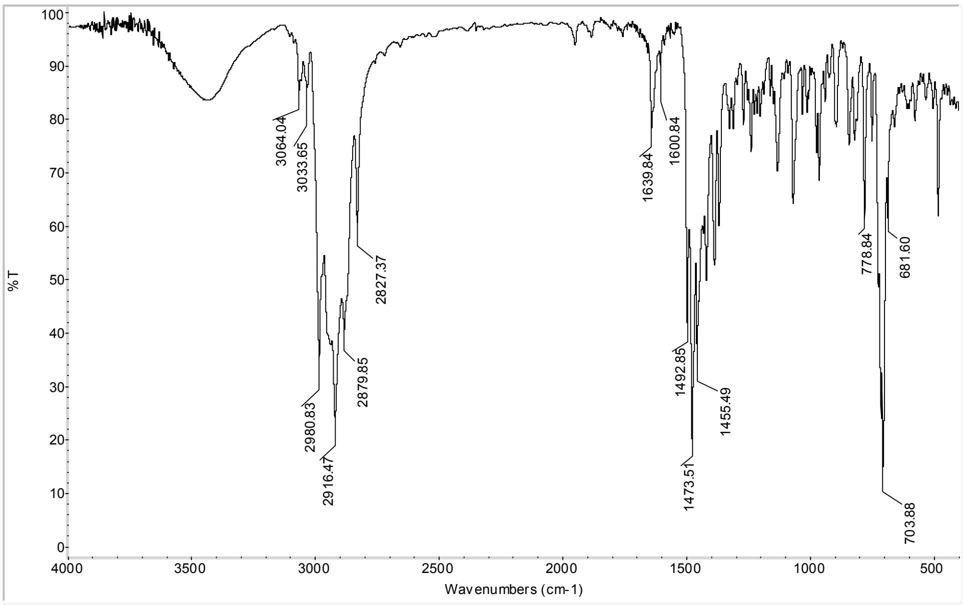


**Figure S41.** FTIR spectrum of the target compound **6e**.

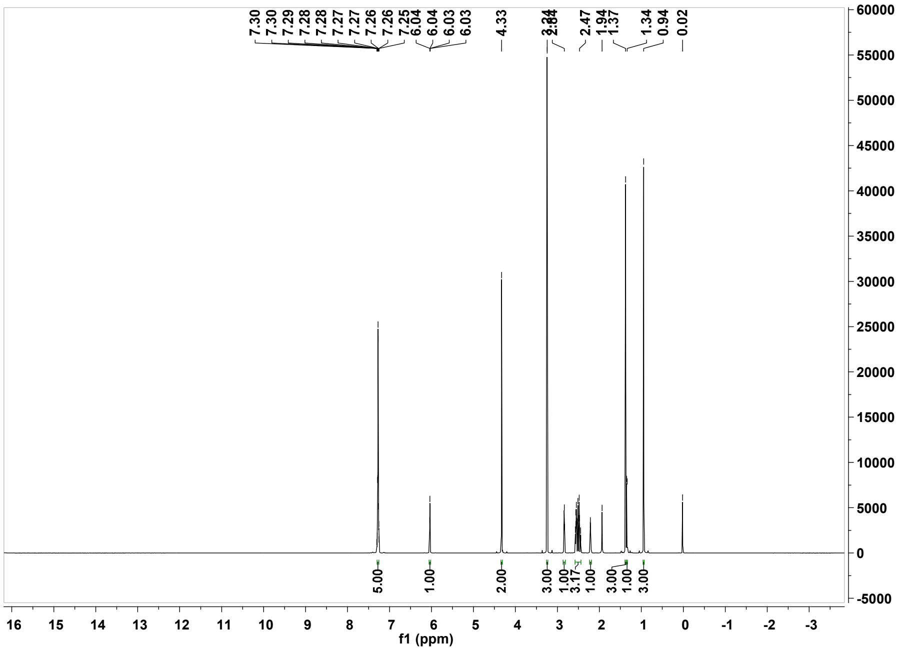


**Figure S42.** $^{1}$H-NMR spectrum of the target compound **6e** in $CDCl_3$.

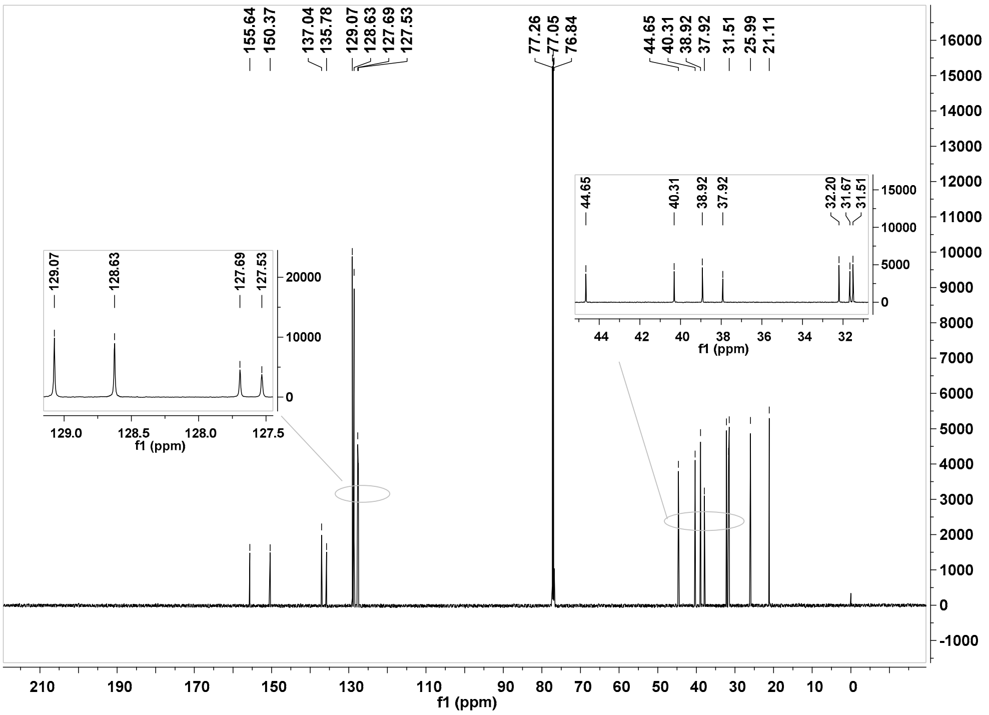


**Figure S43.** $^{13}$C-NMR spectrum of the target compound **6e** in $CDCl_3$.

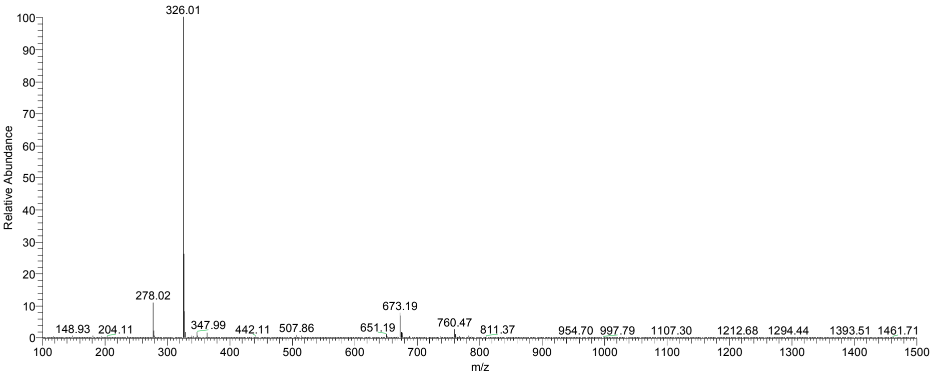


**Figure S44.** ESI-MS spectrum of the target compound **6e**.

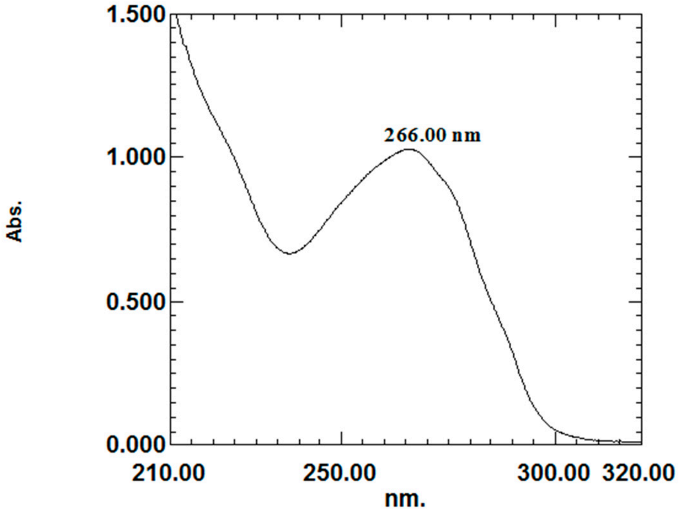


**Figure S45.** UV-vis spectrum of the target compound **6f** in cyclohexane.

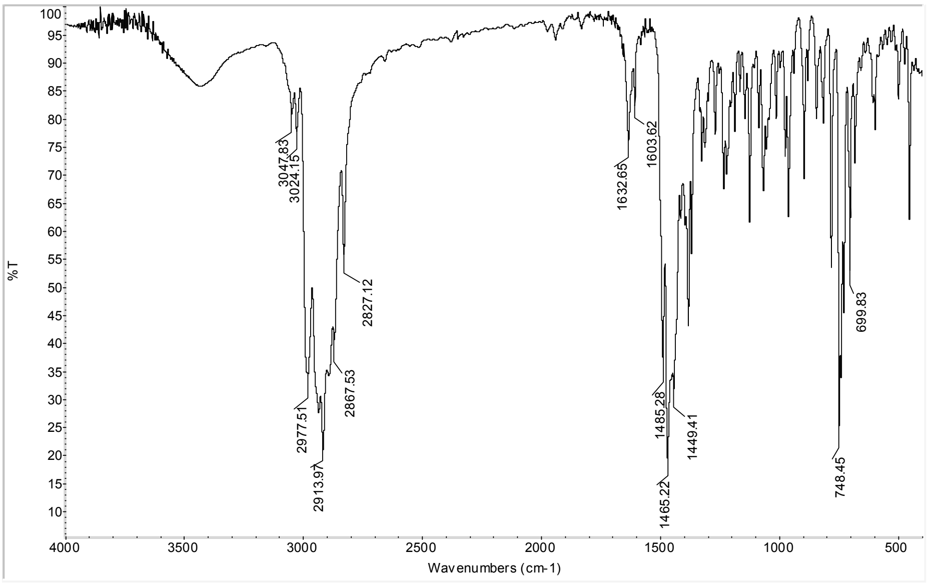


**Figure S46.** FTIR spectrum of the target compound **6f**.

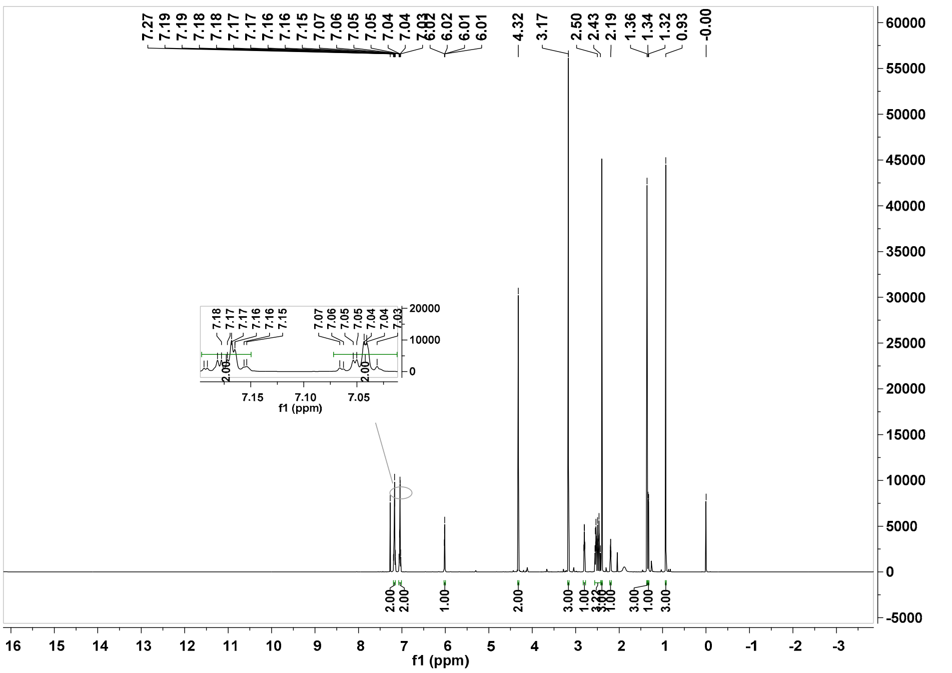


**Figure S47.** $^{1}$H-NMR spectrum of the target compound **6f** in $CDCl_3$.

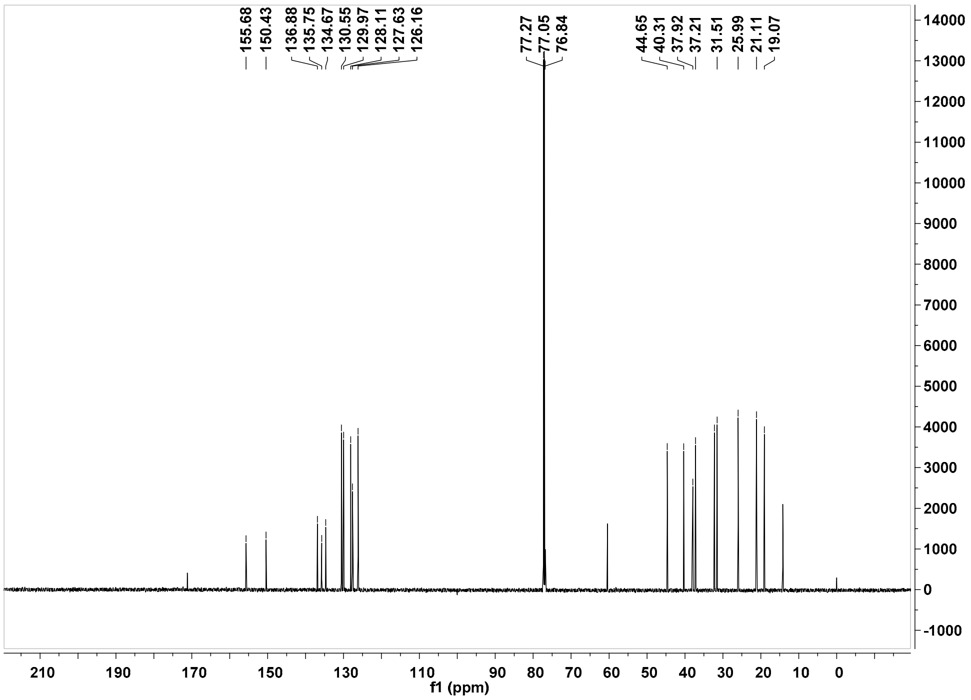


**Figure S48.** $^{13}$C-NMR spectrum of the target compound **6f** in $CDCl_3$.

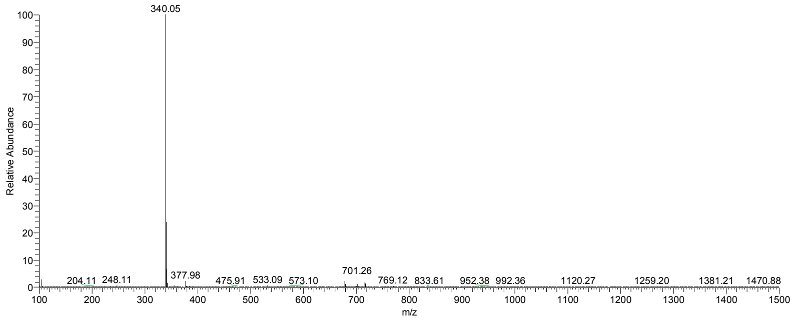


**Figure S49.** ESI-MS spectrum of the target compound **6f**.

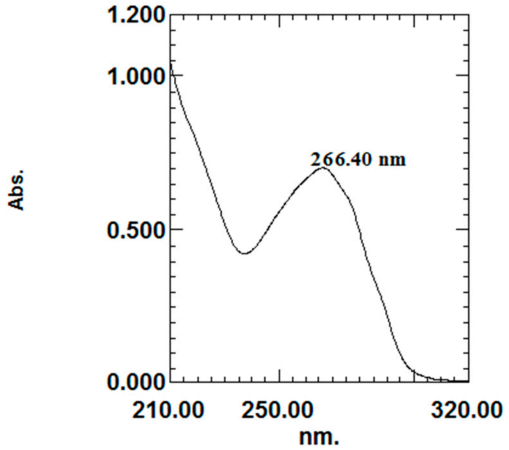


**Figure S50.** UV-vis spectrum of the target compound **6g** in cyclohexane.

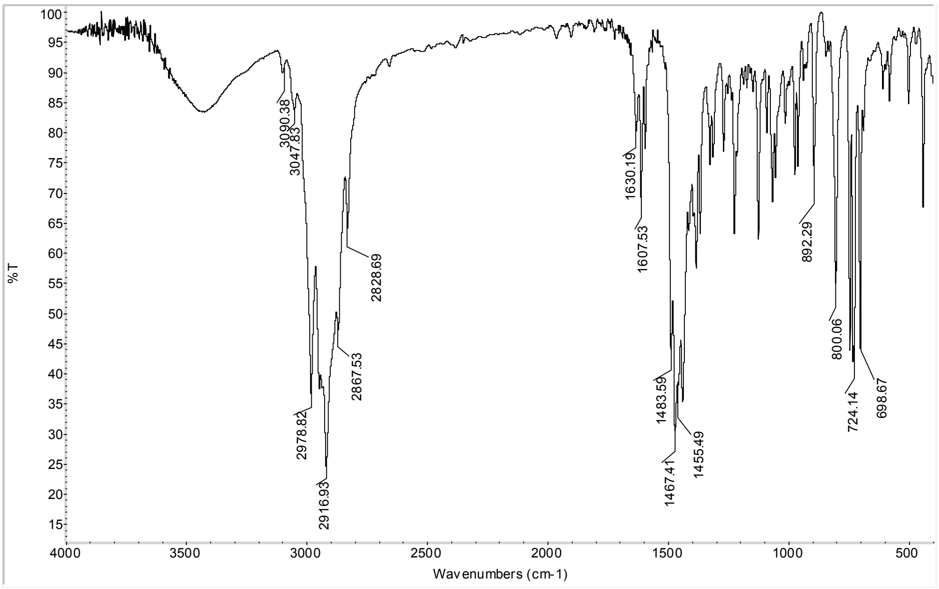


**Figure S51.** FTIR spectrum of the target compound **6g**.

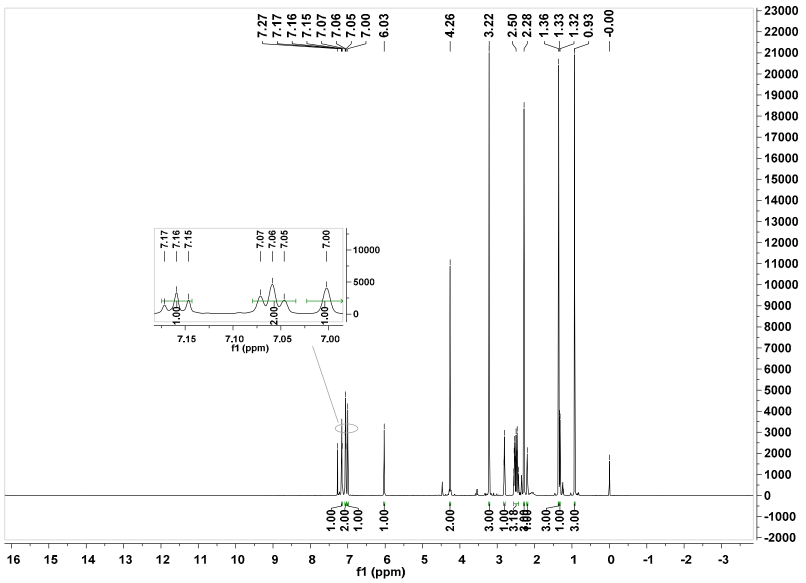


**Figure S52.** $^{1}$H-NMR spectrum of the target compound **6g** in $CDCl_3$.

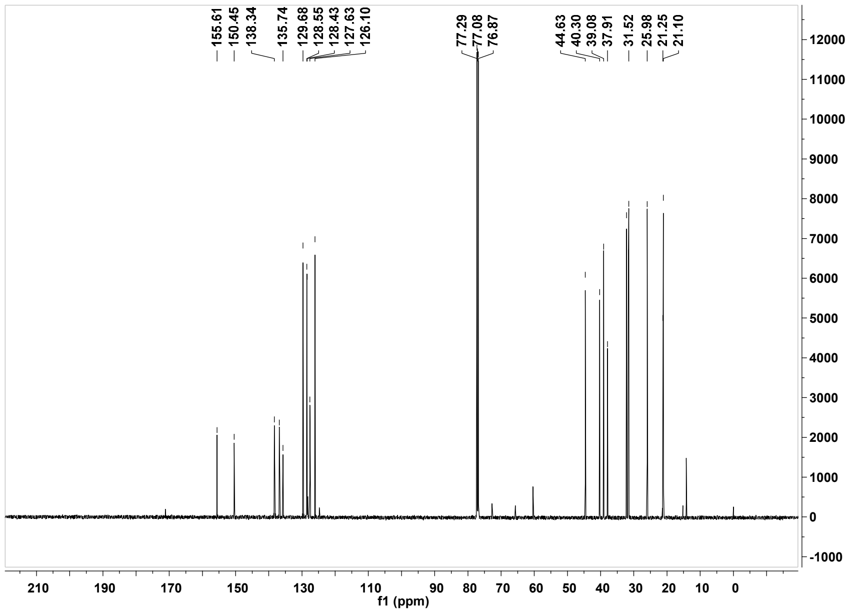


**Figure S53.** $^{13}$C-NMR spectrum of the target compound **6g** in $CDCl_3$.

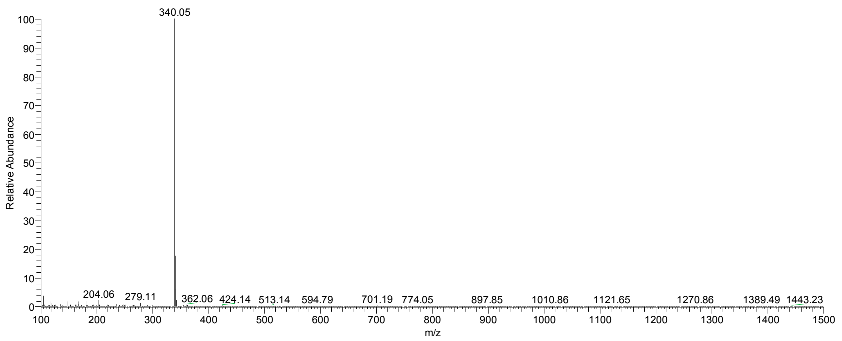


**Figure S54.** ESI-MS spectrum of the target compound **6g**.

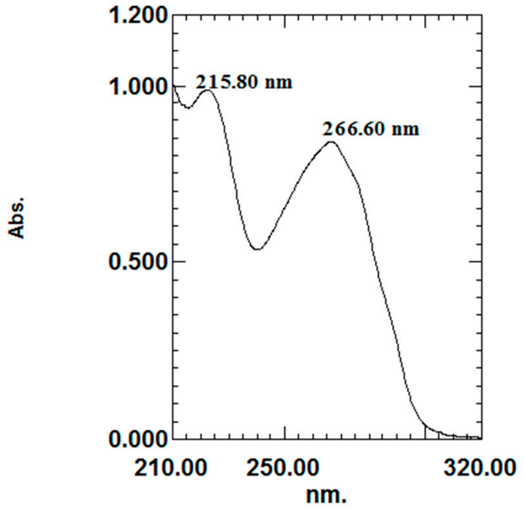


**Figure S55.** UV-vis spectrum of the target compound **6h** in cyclohexane.

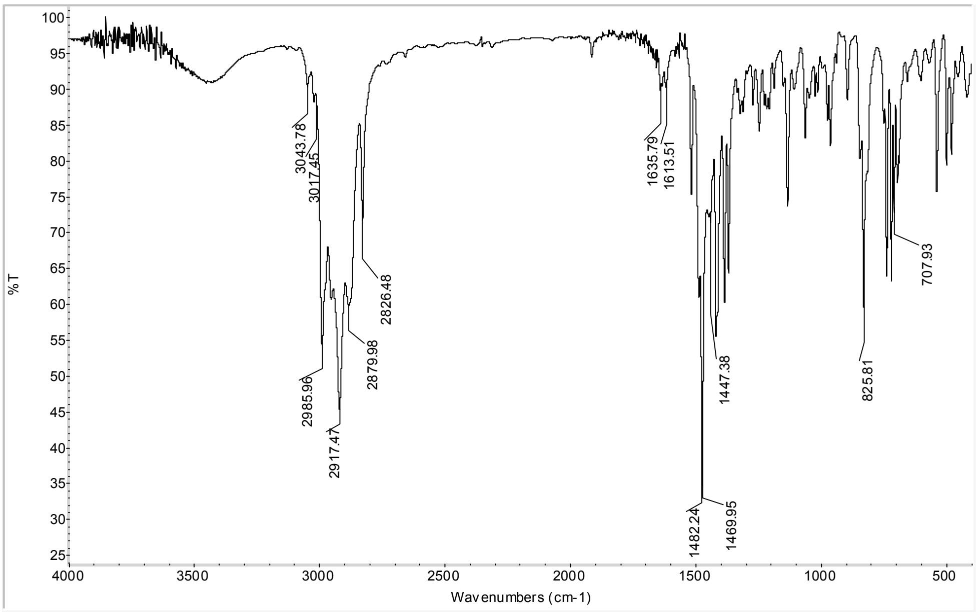


**Figure S56.** FTIR spectrum of the target compound **6h**.

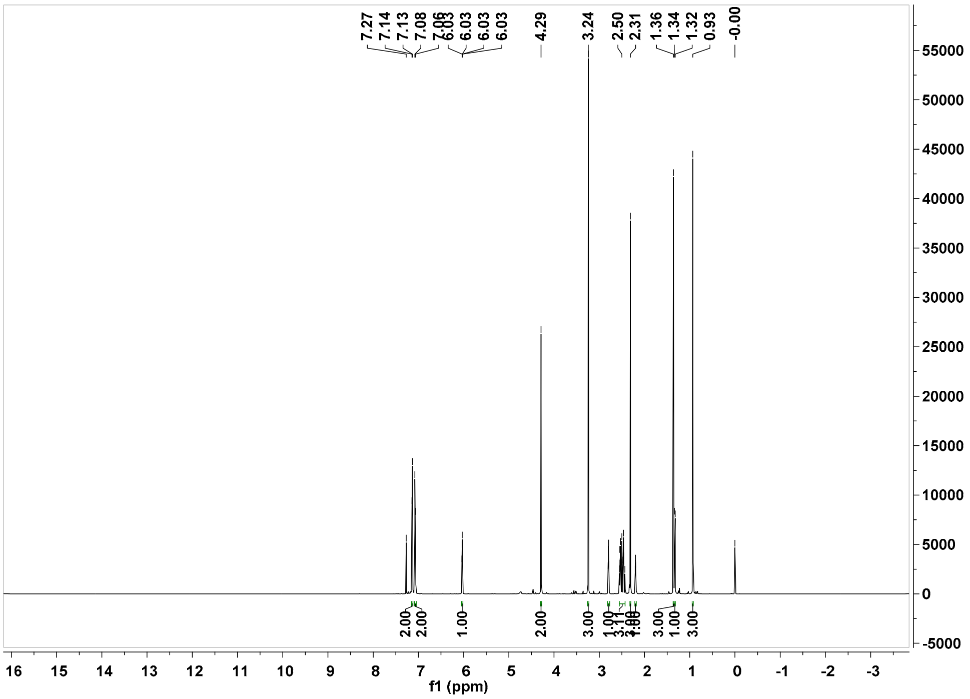

**Figure S57.** $^{1}$H-NMR spectrum of the target compound **6h** in $CDCl_3$.

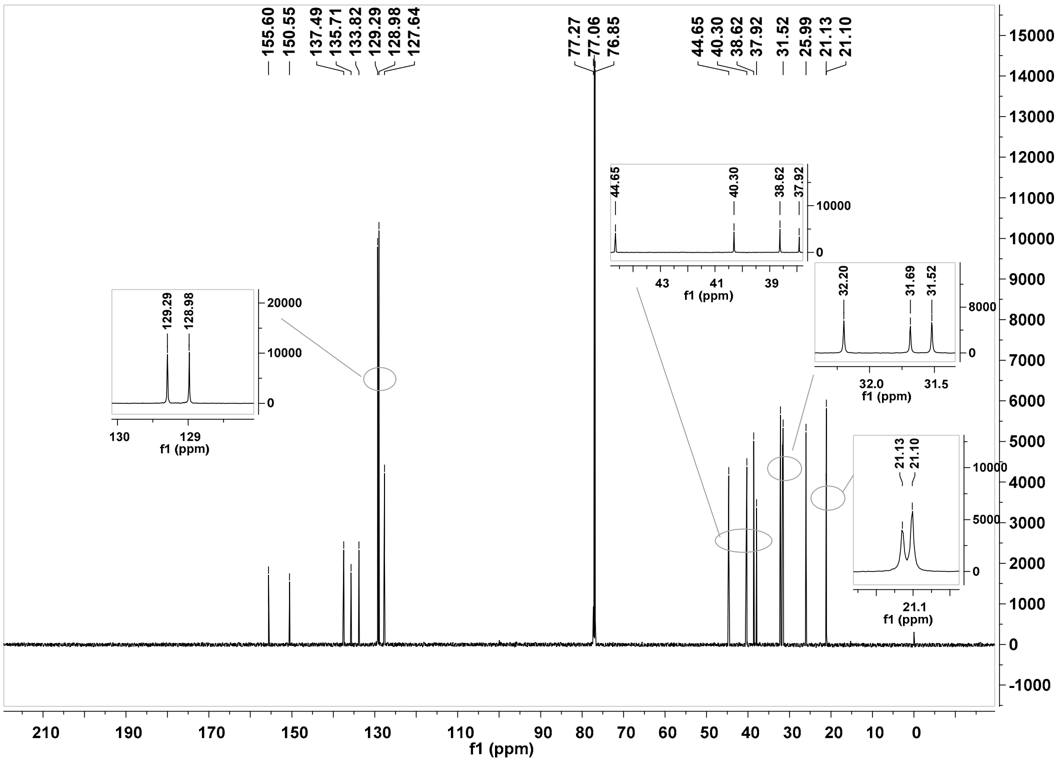

**Figure S58.** $^{13}$C-NMR spectrum of the target compound **6h** in $CDCl_3$.

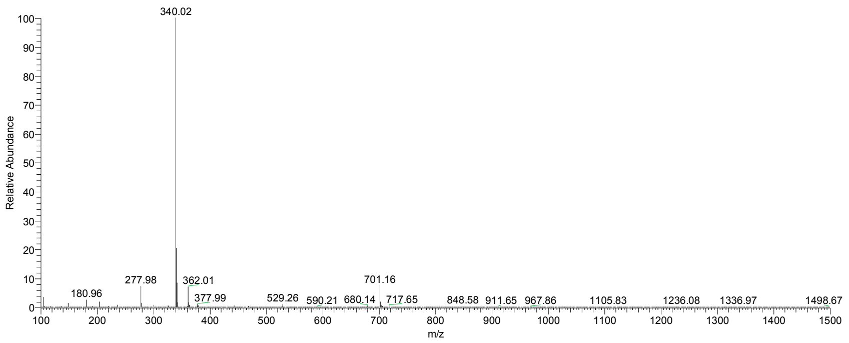


**Figure S59.** ESI-MS spectrum of the target compound **6h**.

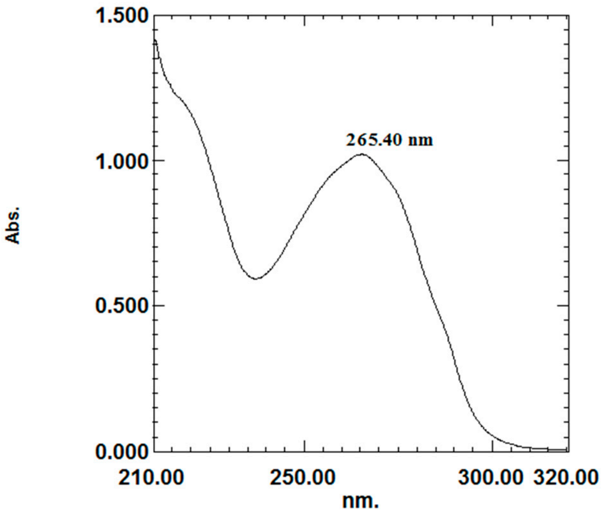


**Figure S60.** UV-vis spectrum of the target compound **6i** in cyclohexane.

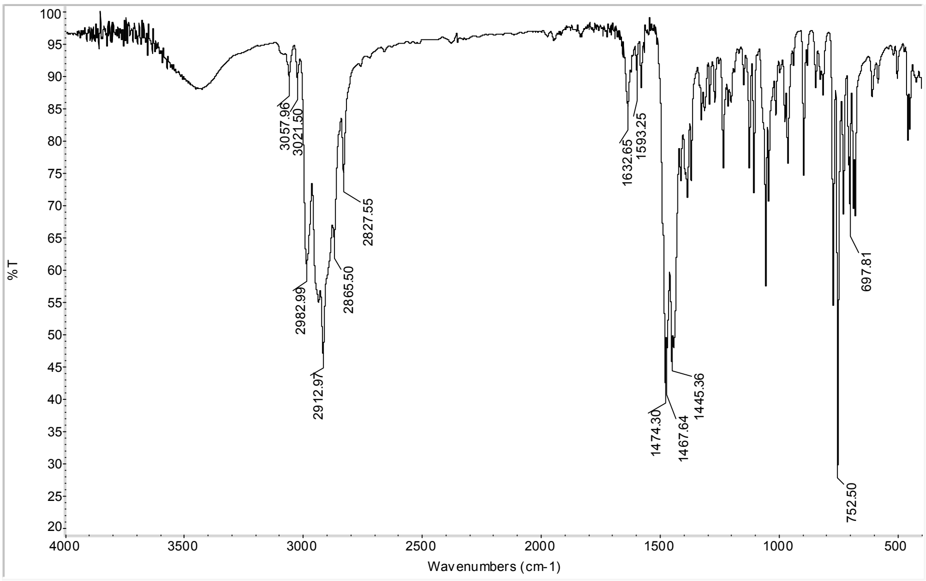


**Figure S61.** FTIR spectrum of the target compound **6i**.

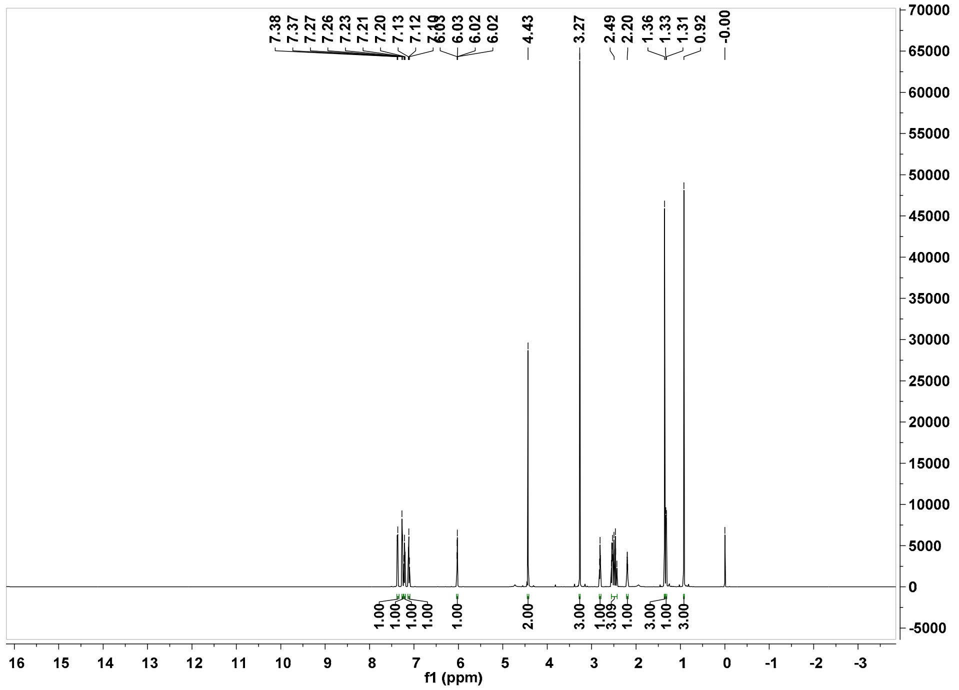


**Figure S62.** $^{1}H$-NMR spectrum of the target compound **6i** in $CDCl_3$.

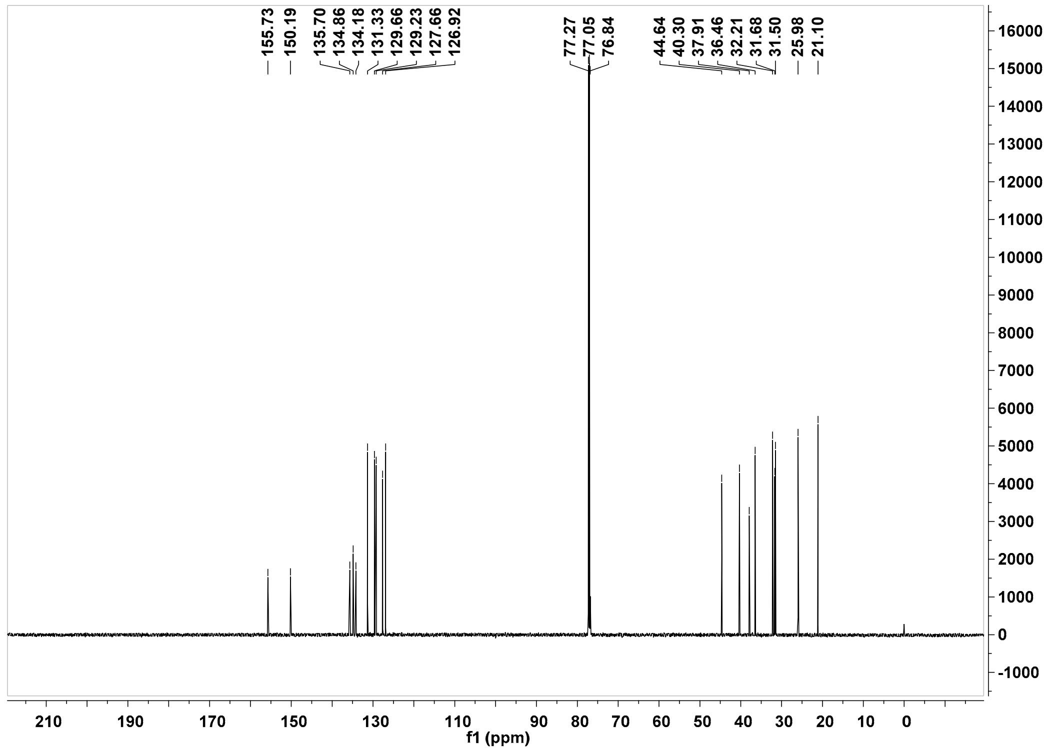


**Figure S63.** $^{13}C$-NMR spectrum of the target compound **6i** in $CDCl_3$.

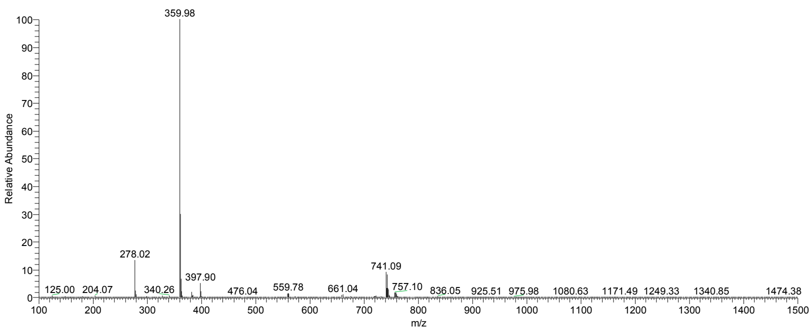


**Figure S64.** ESI-MS spectrum of the target compound **6i**.

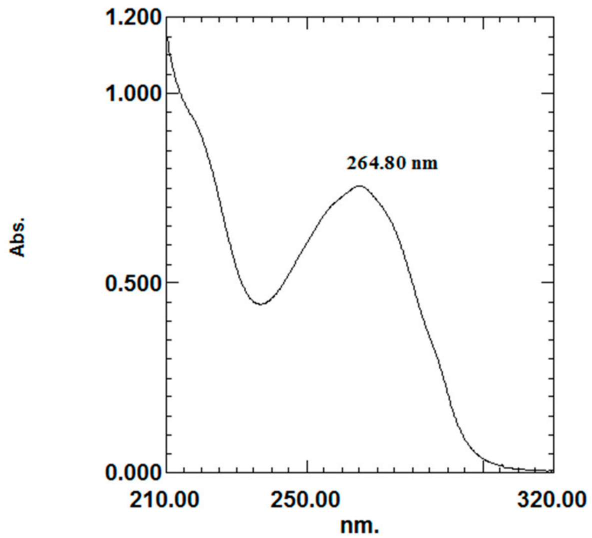


**Figure S65.** UV-vis spectrum of the target compound **6j** in cyclohexane.

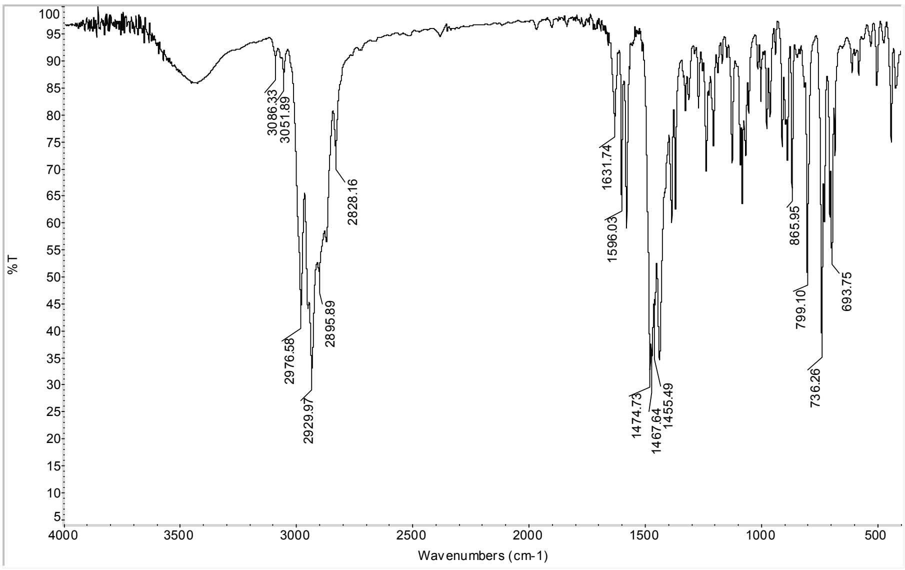


**Figure S66.** FTIR spectrum of the target compound **6j**.

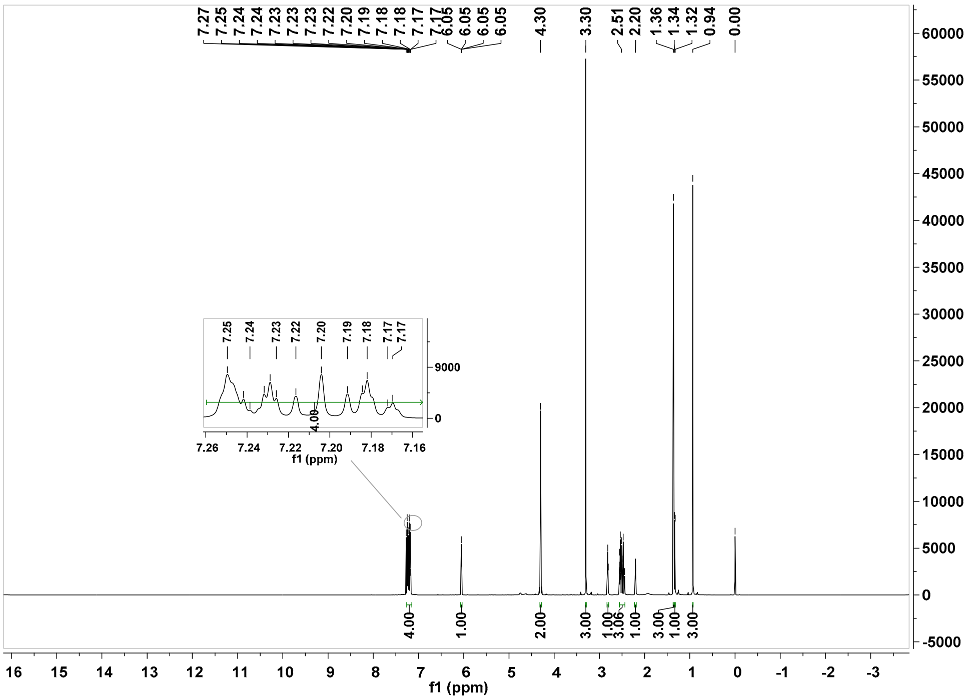


**Figure S67.** $^{1}$H-NMR spectrum of the target compound **6j** in $CDCl_3$.

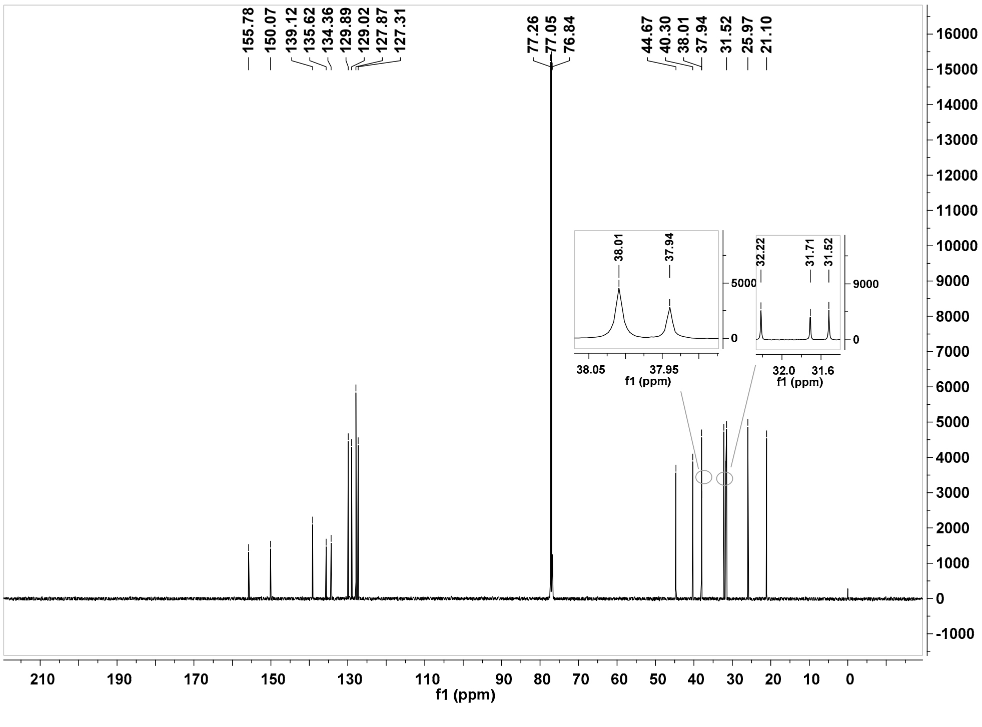


**Figure S68.** $^{13}$C-NMR spectrum of the target compound **6j** in $CDCl_3$.

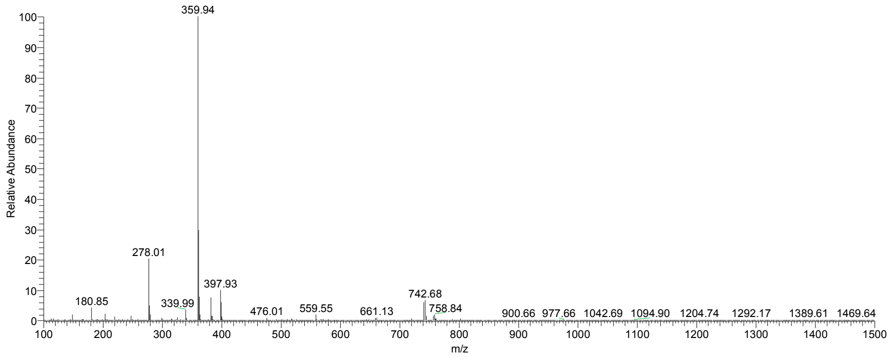


**Figure S69.** ESI-MS spectrum of the target compound **6j**.

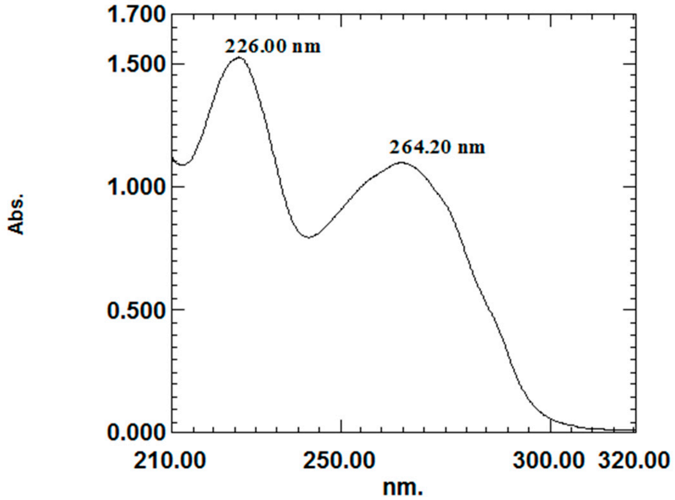


**Figure S70.** UV-vis spectrum of the target compound **6k** in cyclohexane.

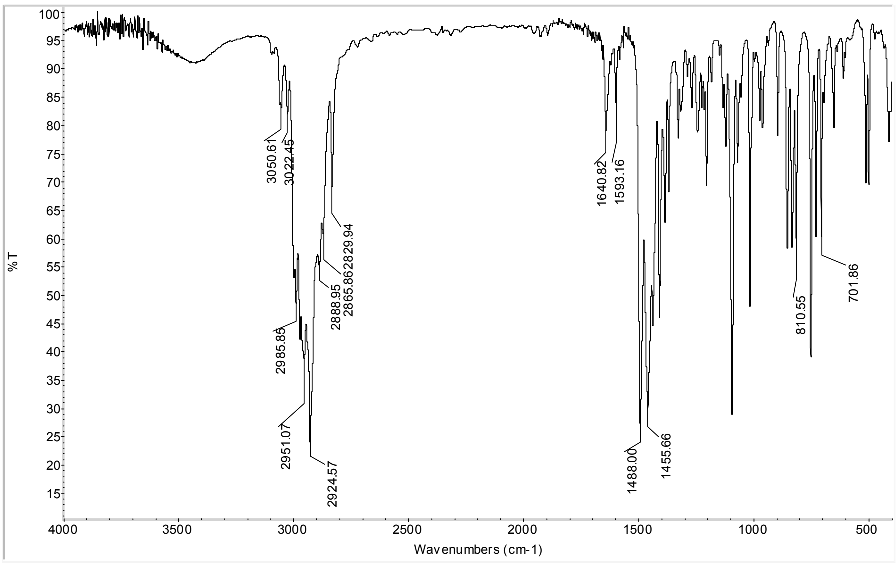


**Figure S71.** FTIR spectrum of the target compound **6k**.

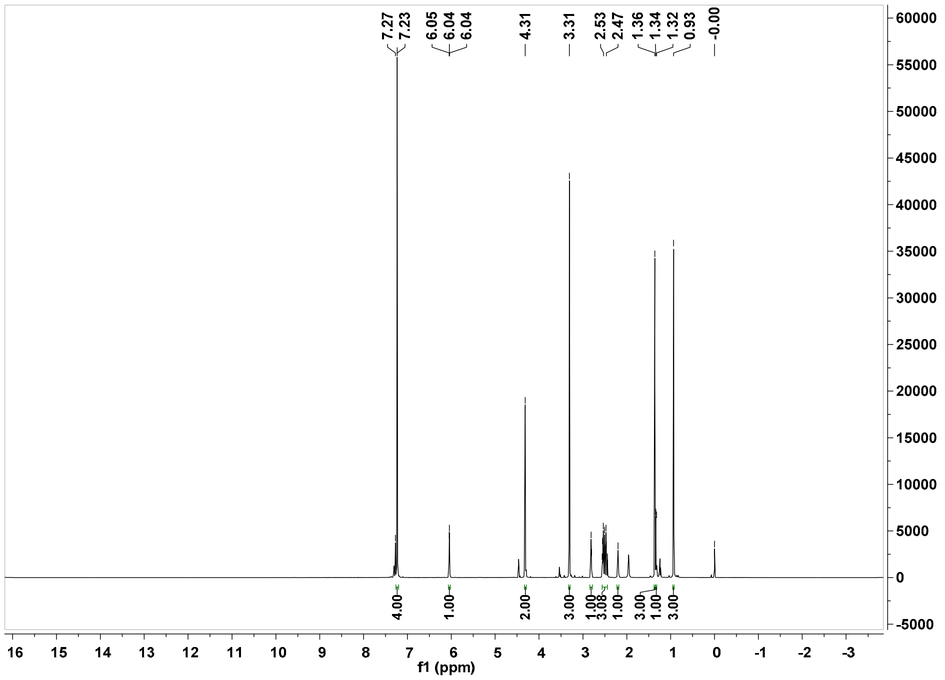


**Figure S72.** $^{1}$H-NMR spectrum of the target compound **6k** in $CDCl_3$.

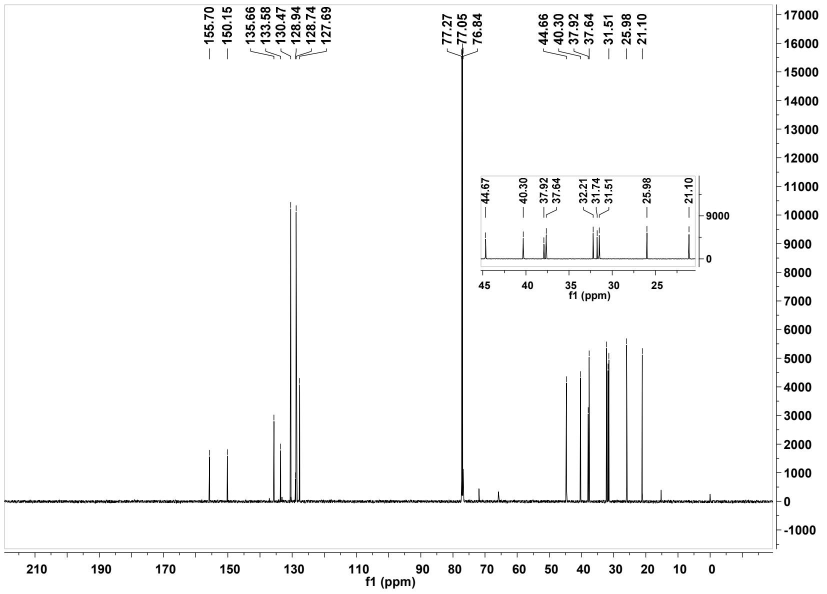


**Figure S73.** $^{13}$C-NMR spectrum of the target compound **6k** in $CDCl_3$.

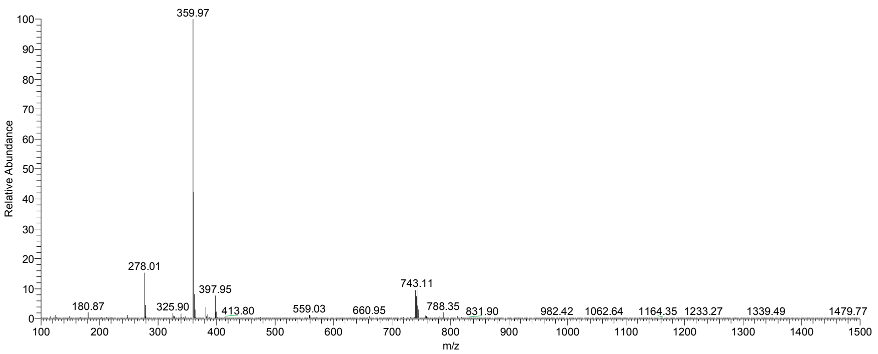

**Figure S74.** ESI-MS spectrum of the target compound **6k**.

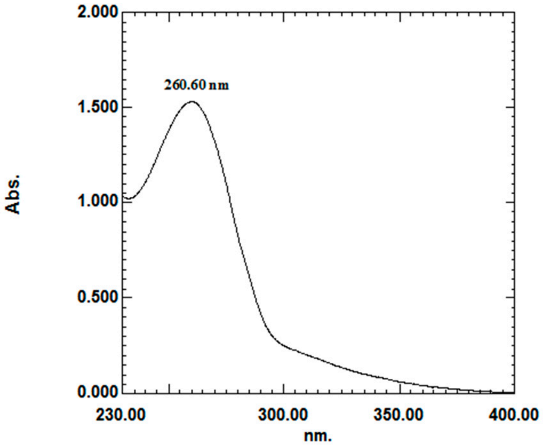


**Figure S75.** UV-vis spectrum of the target compound **6l** in EtOH.

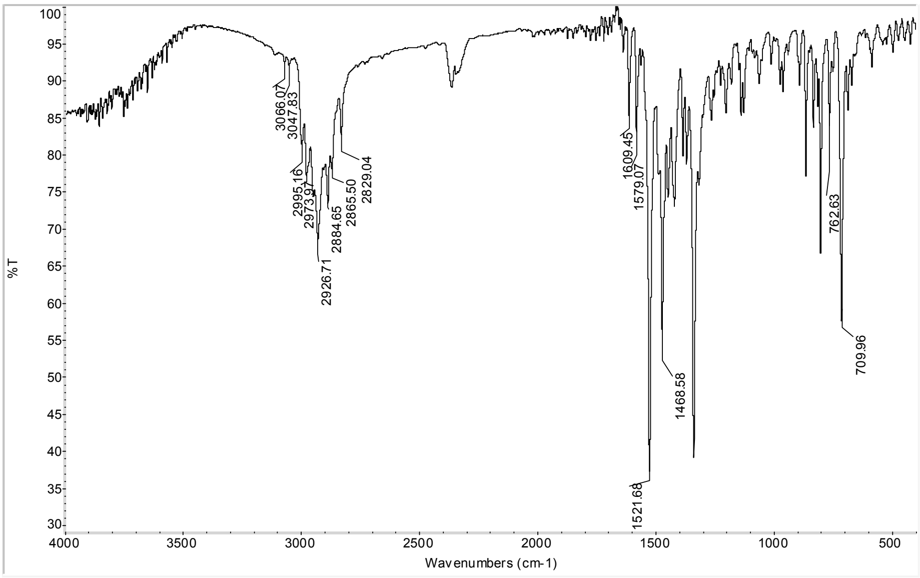


**Figure S76.** FTIR spectrum of the target compound **6l**.

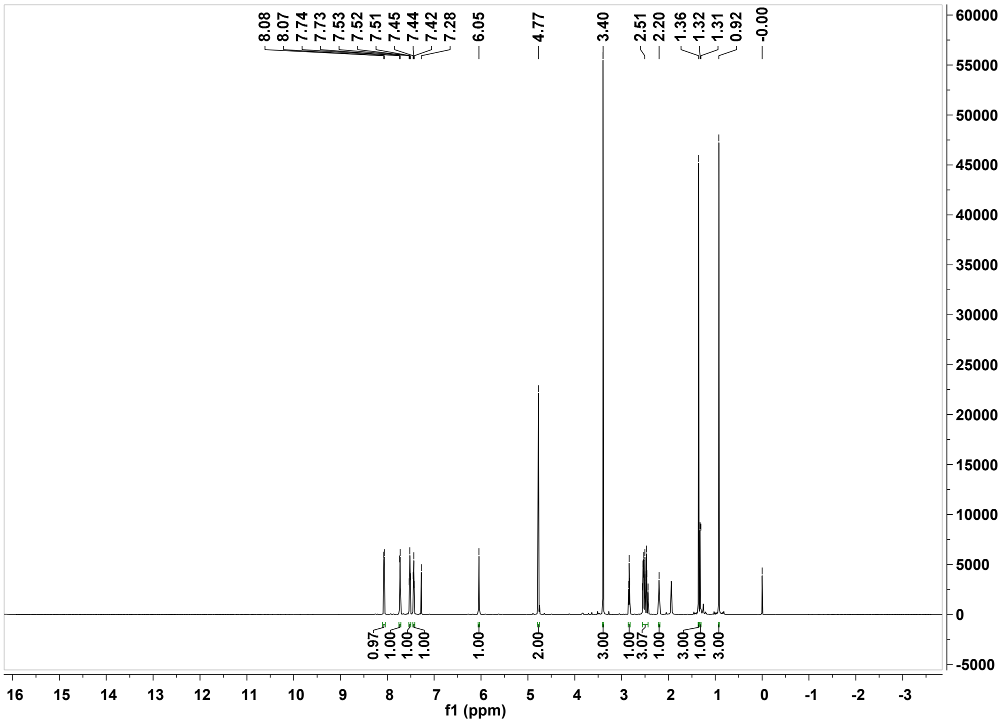

**Figure S77.** $^{1}$H-NMR spectrum of the target compound **6l** in $CDCl_3$.

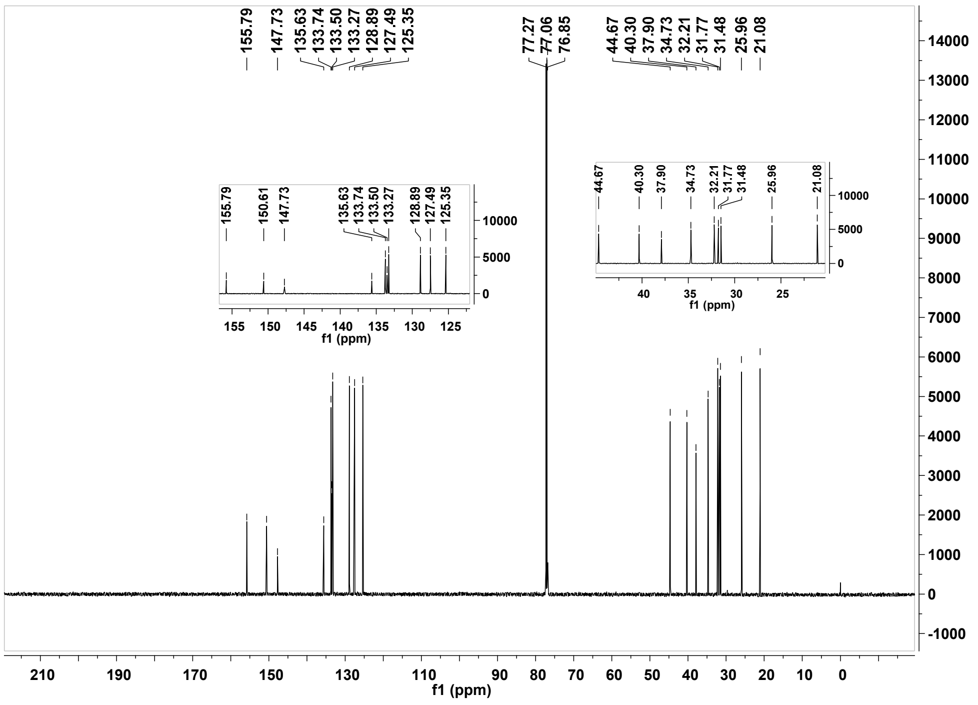

**Figure S78.** $^{13}$C-NMR spectrum of the target compound **6l** in $CDCl_3$.

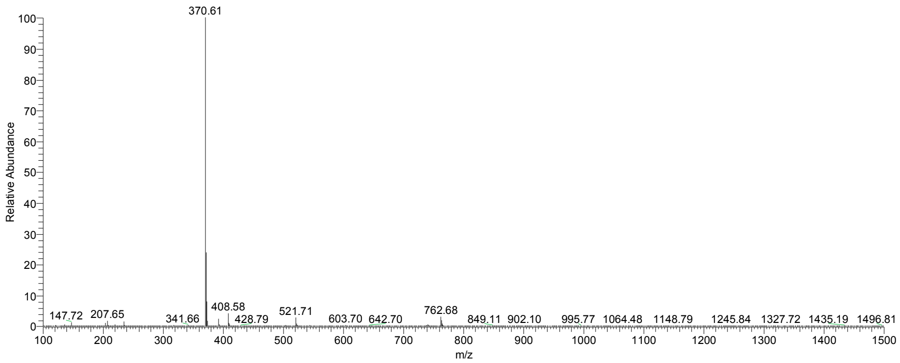


**Figure S79.** ESI-MS spectrum of the target compound **6l**.

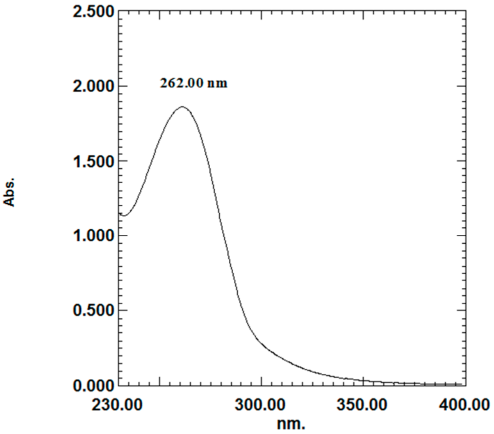


**Figure S80.** UV-vis spectrum of the target compound **6m** in EtOH.

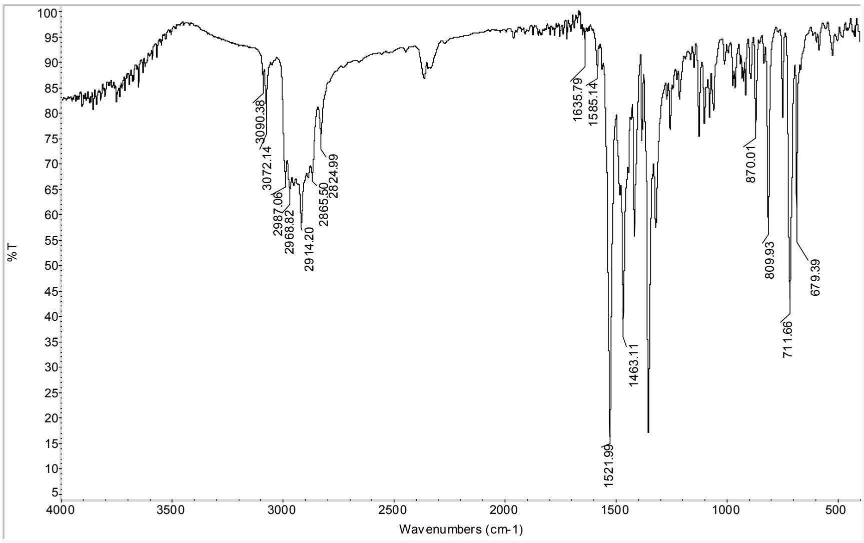


**Figure S81.** FTIR spectrum of the target compound **6m**.

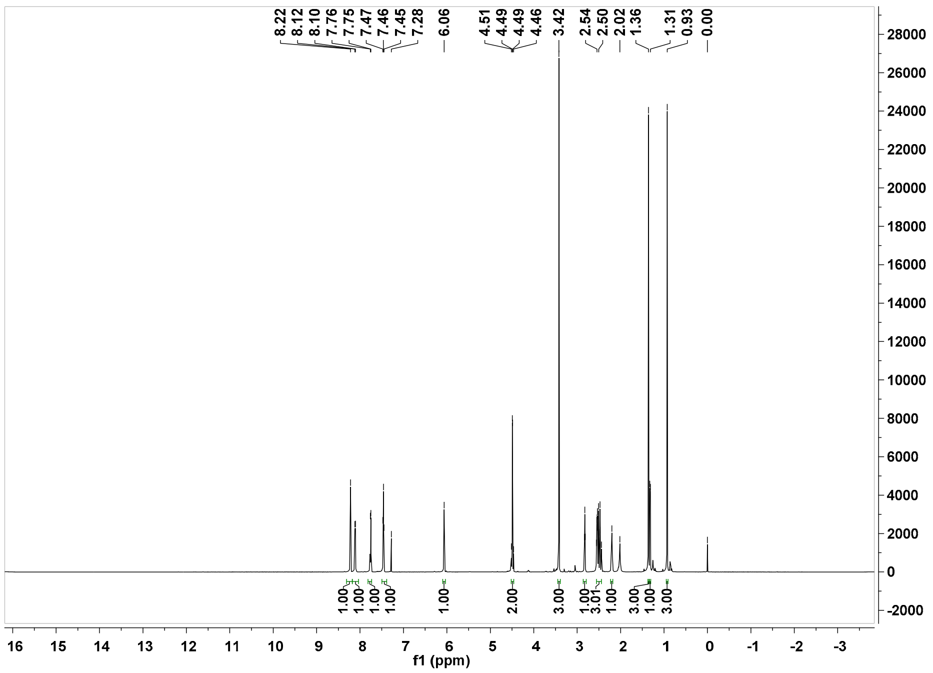


**Figure S82.** $^{1}H$-NMR spectrum of the target compound **6m** in $CDCl_3$.

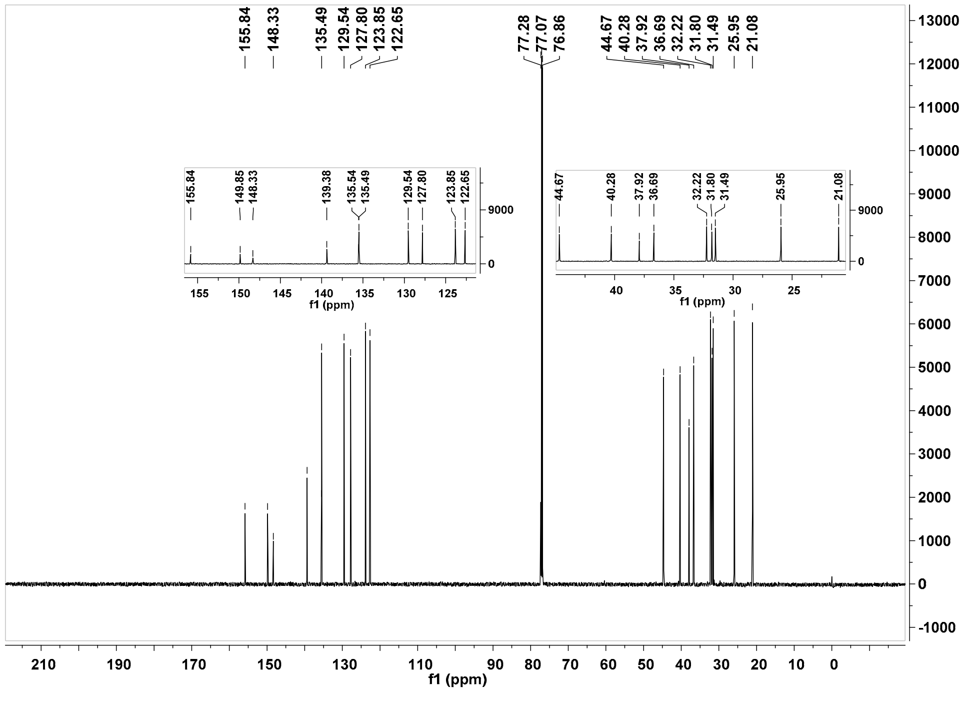


**Figure S83.** $^{13}C$-NMR spectrum of the target compound **6m** in $CDCl_3$.

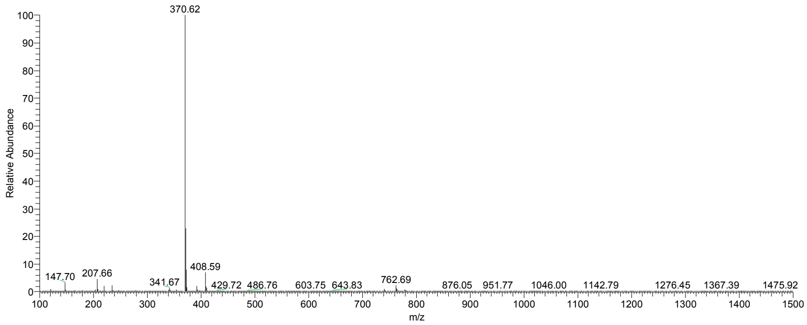


**Figure S84.** ESI-MS spectrum of the target compound **6m**.

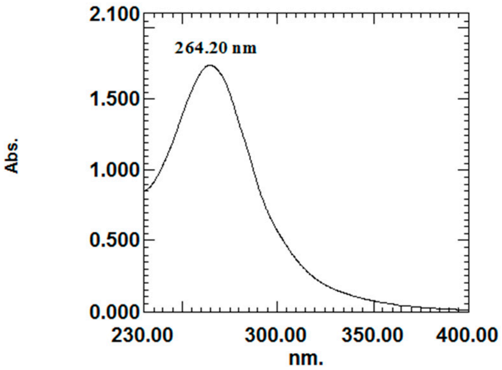


**Figure S84.** UV-vis spectrum of the target compound **6n** in EtOH.

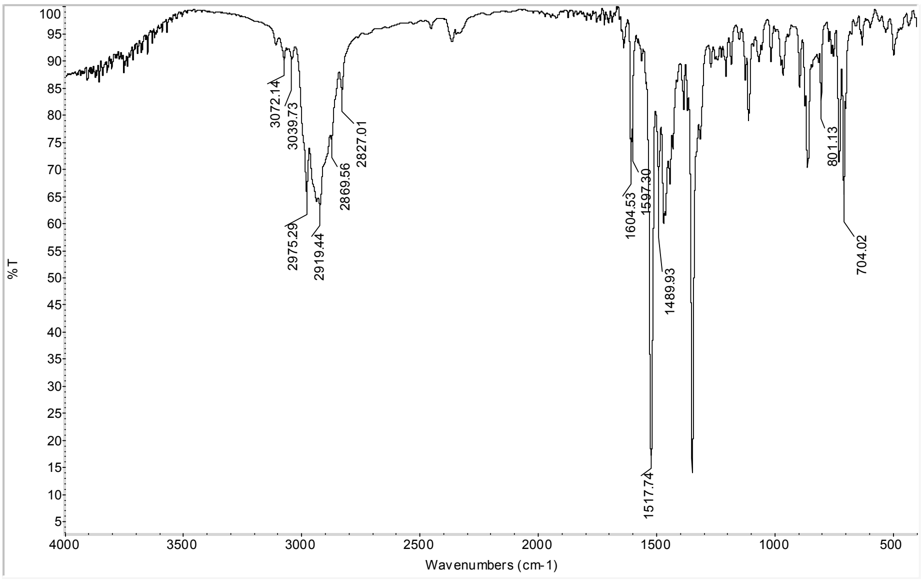


**Figure S86.** FTIR spectrum of the target compound **6n**.

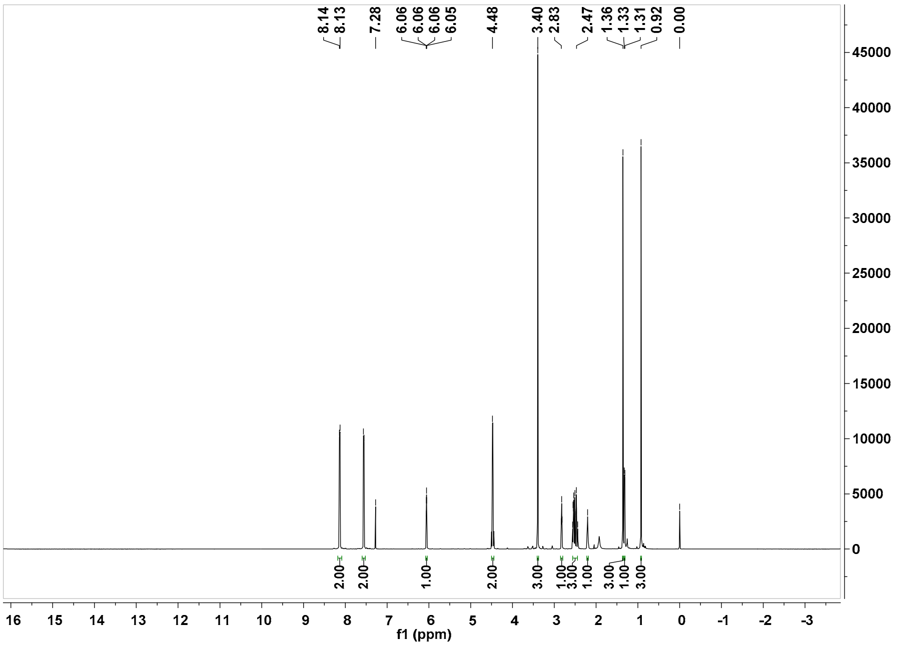

**Figure S87.** $^{1}H$-NMR spectrum of the target compound **6n** in $CDCl_3$.

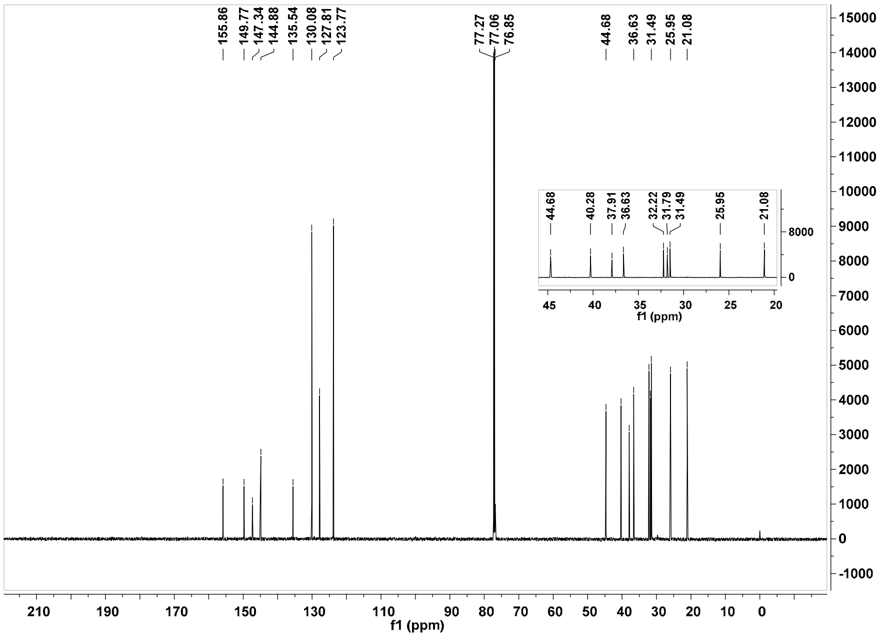

**Figure S88.** $^{13}C$-NMR spectrum of the target compound **6n** in $CDCl_3$.

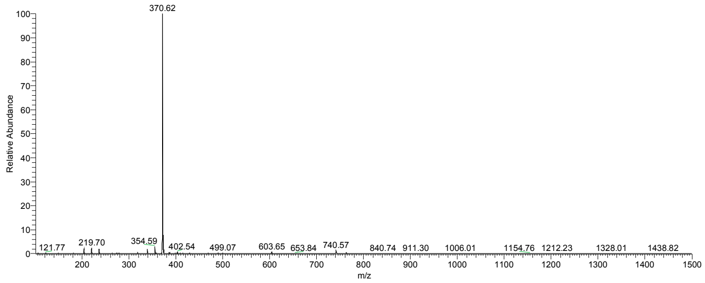

**Figure S89.** ESI-MS spectrum of the target compound **6n**.
